# Comparative analysis of flower volatiles from four *Jasminum* species growing in Egypt using multivariate analysis

**DOI:** 10.1038/s41598-026-39688-w

**Published:** 2026-03-11

**Authors:** Mohamed S. Yassen, Iriny M. Ayoub, Sherweit H. El-Ahmady, Abdel Nasser B. Singab

**Affiliations:** 1 Department of Pharmacognosy, Faculty of Pharmacy, Ain Shams University, Abbassia, 11566 Cairo Egypt; 2 Center for Drug Discovery Research and Development, Faculty of Pharmacy, Ain Shams University, Abbassia, 11566 Cairo Egypt

**Keywords:** Jasminum, Antidepressant activity, MAO-A inhibition, GC-MS-headspace, Multivariate analysis, Natural variation in plants, Secondary metabolism, Plant sciences

## Abstract

The genus *Jasminum* is a valuable oil-bearing shrub in the *Oleaceae* family that produces concrete and absolute jasmine scents. Jasmine is an important floral ingredient in fragrances, soaps, cosmetics, and toiletries. As an export commodity, it is important to establish effective analytical procedures for authenticating jasmine species and detecting adulterants. In the current investigation, the volatile constituents of the concrete and absolute of *Jasminum sambac* (L.) Aiton, *J. azoricum* L., *J. grandiflorum* L., *J. multiflorum* (Burm. f.) Andrews harvested in August, in addition to *J. grandiflorum* factory products were analyzed using Gas Chromatography-Mass Spectrometry (GC-MS). Furthermore, Headspace-GC-MS (HS-GC-MS) was used to investigate variations in the volatile oil composition of the flowers of the four *Jasminum* species throughout June to August. A total of 157 volatile components were identified belonging to various classes including monoterpene hydrocarbons, oxygenated monoterpenes, sesquiterpene hydrocarbons, oxygenated sesquiterpenes, diterpenes, triterpenes, phenylpropanoids/benzenoids, fatty-acid derivatives, aliphatic hydrocarbons, and nitrogenous compounds. Unsupervised multivariate data analysis tools, including Principal Component Analysis (PCA) and Hierarchical Cluster Analysis (HCA), were used to distinguish between the oil complexity within parameters such as species and seasonal variation. Moreover, the inhibitory effects of the concrete and absolute on monoamine oxidase A (MAO-A) were evaluated in vitro. Concrete and absolute showed a potent MAO-A inhibitory activity with half-maximal inhibitory concentrations (IC_50_) ranging from 4 to 35.39 µg/mL and 0.46 to 15.82 µg/mL, respectively. This study showed that jasmine blooms′ volatiles displayed promising in vitro MAO-A inhibitory activity, which establishing a biochemical basis for future mechanistic investigations.

**Supplementary Information:**

The online version contains supplementary material available at 10.1038/s41598-026-39688-w.

## Introduction

*Jasminum* is a significant genus in the olive family (*Oleaceae*) with over 200 species found worldwide. Jasmine is currently widely cultivated in tropical, subtropical, and temperate zones across the world as a fragrant plant, and its blossoms are used to make essential oil and jasmine tea^1,2^. *J. grandiflorum* is grown in considerable amounts, mostly in India and Egypt, and to a smaller extent in Morocco and South Africa^3^. The primary producer of *J. grandiflorum* concrete and absolute is Egypt^4–6^. The jasmine flower starts to thrive in warm climates and throughout the summer. In Egypt, the flower starts to bloom usually in April and reaches the peak of the blooming season in July, and decreases in September^7^. The best aroma comes from flowers that open at night^8^. The fully bloomed flower is harvested for its oil and concrete. To “wash” the volatile compounds out of the flowers, a non-polar solvent like hexane is used. “Concrete” is the waxy, semi-solid substance extracted after hexane evaporation. Following that, the concrete goes through multiple rounds of “washings” using a solvent that is polar, like ethanol. Ethanol’s polarity will enable the extraction of concrete volatile aromatics while leaving behind the non-polar plant waxes that are not dissolved in ethanol. Ethanol is finally evaporated, leaving the “Absolute” behind^4^. Approximately 10,000 flowers are needed to produce one kilogram of concrete jasmine^4^.

In the current study, four *Jasminum* species were selected owing to their economic and aromatic value in the fragrance and pharmaceutical industries, besides, their availability in Egypt. *Jasminum grandiflorum* Linn. is often known as Royal jasmine, Spanish jasmine, and Catalonian jasmine^9^. It is indigenous to China, the Philippines, Persia, India, and Afghanistan^10^. *Jasminum sambac* (L.) Ait. is a shrub with white blooms that differ from *J. grandiflorum* L. in volatile compounds. The blossom’s petals are waxier and thicker, and the shrub’s stem is thicker than *J. grandiflorum*. *Jasminum multiflorum* (Burm. f.) Andrews, also known as Star jasmine, Furry jasmine, Downy jasmine, and Pinwheel jasmine, has entire white blooms in clusters that are quite attractive and bloom freely for a long time. The blooms are sessile, aromatic, with a faint pink bud, a pure white opening flower, and growing in terminal umbels; petals 6 to 9, lanceolate in shape^11^. *Jasminum azoricum* L., or the white Azorean jasmine, is a rare but valued ornamental shrub native to the island of Madeira that has been recognized since the late seventeenth century^12^. It is a climbing shrub with trifoliate leaves and fragrant flowers that grow in clusters of 1–5 at the tip of branches. The blooms have a little green calyx and a white tubular corolla that expands into 6 petal lobes^13^.

The volatile components of jasmine tea prepared from *J. sambac* flowers, a commonly consumed beverage, have been studied^14^. Previous studies showed that the seven main volatile components of Chinese jasmine tea prepared by continuously mixing oils of *J. sambac* flowers with base tea were methyl anthranilate, indole, *α*-farnesene, benzyl alcohol, linalool, benzyl acetate, and (*Z*)-3-hexenyl benzoate employing solid-phase microextraction (SPME)^15^. Benzyl acetate, (*E*,* E*)-*α* -farnesene, linalool, methyl anthranilate, and *cis*-3-hexenyl acetate were the major volatile constituents detected in *J. sambac* from Egypt and India^7,16,17^. Additionally, benzyl acetate and linalool are the chief scent volatiles *in J. grandiflorum* concrete and absolute from Egypt and India analyzed by SPME^8,16^.

The essential oil and the flower extract of *J. grandiflorum* have shown efficacy as antiviral^18^, antibacterial^19,20^, aphrodisiac^10^, anti-inflammatory^21,22^, antioxidant^10^, anti-hypertensive^10^, anthelmintic^23^, and antidiabetic^24^. They have been used to treat toothache^10^, ringworm infection, ulcers^10^, stomatitis^10^, skin problems^10^and for wound healing^25–27^. Furthermore, *J. sambac* flower has been used to treat diarrhea, stomach discomfort, conjunctivitis, and dermatitis^1^.

Research indicated that the main ingredient, benzyl acetate, was reported in the literature to be associated with biological activities relevant to neurodegenerative disorders, including Alzheimer’s and Parkinson’s disease^28^. This substance’s neuroprotective effect may stem from its capacity to regulate neurotransmitter function and reduce oxidative damage. Linalool and indole, found in small amounts in extracts of *Jasminum officinale* L., have been previously reported to modulate neurotransmitter activity^28^. Additionally, experiments on animal models have shown that the essential oil of *J. sambac* may potentially produce anxiolytic activity, and animal movement was considerably decreased^29^. Besides, eugenol, farnesol, nerolidol, and phytol, key components of jasmine concrete and absolute, have been noted for their neuroprotective, anti-inflammatory, and antioxidant effects^30–34^.

Depression is a common mental condition that affects over 17% of adults, with a greater prevalence in women (10–30% vs. 7–15% in males)^35^. Depression is believed to be caused by a deficiency of monoamines, according to the monoamine theory. Pharmacological interventions aimed at increasing monoamine availability. Selective serotonin reuptake inhibitors (SSRIs), selective norepinephrine reuptake inhibitors (SNRIs), and monoamine oxidase inhibitors (MAOIs), have demonstrated efficacy in 60–70% of patients^36^. However, current antidepressant drugs have limitations, including limited activity, significant side effects, slow onset of action, and poor compliance^37–39^. Thus, the pursuit for novel medications to treat severe depression remains ongoing^38,40^. Recent research suggests that plant and dietary extracts might inhibit MAO enzymes, leading to protection from neurotoxins and oxidative stress^41–43^.

The main enzyme that catalyzes the oxidative deamination of amines and neurotransmitters that contribute to mood disorders, depressive disorders, oxidative stress, and adverse medication reactions is called monoamine oxidase (MAO)^42^. MAO degrades monoamines such as 5-hydroxytryptamine (5-HT), histamine, and catecholamines, including dopamine, noradrenaline, and adrenaline^44^. There are two distinct isoforms of mitochondrial FAD-containing enzymes known as MAOs: MAO-A and MAO-B. These isoforms differ in both structure and function. Different ratios of both isoforms are expressed in all tissues. Although both types of isozymes are present in the kidney, liver, and brain, MAO-A is mostly expressed in the heart, skin fibroblast, and adipose tissue, while MAO-B is primarily found in platelets and lymphocytes^45–47^. MAO-B has a role in neurodegenerative illnesses, whereas MAO-A contributes to mental problems, including depression. MAO-B inhibitors provide neuroprotection, whereas MAO-A inhibitors are excellent antidepressants in both human and animal models but may cause adverse effects, such as hypertension, when combined with tyramine-containing meals^44,48^.

Aromatherapy has been shown to alleviate anxiety, stress, depression and increase overall well-being. For example, the volatile oil of *J. grandiflorum* is a source of the herbal stress hormone methyl jasmonate (MJ)^49^. Furthermore, a prior study has shown that MJ exhibited antidepressant effects^50^. Aromatherapy using Jasmine Essential Oil (JEO) may alleviate preoperative anxiety in patients having laparotomy^51^. Furthermore, inhalation of *J. sambac* essential oil can improve sleep quality and duration in haemodialysis patients^52^. (*E*,* E*)-*α*-Farnesene, one of the primary components of jasmine, was reported to possess MAO-A enzyme inhibitory activity^53^. In addition, previous studies had shown that *J. grandiflorum* extracts exhibited greater MAO-A inhibitory activity in comparison to the reference standard, clorgyline, indicating its antioxidant potential to minimize cell oxidative damage and reduce symptoms of depression^54^.

The purpose of this study was to determine the compositional differences of volatile compounds from *J. sambac*, *J. azoricum*, *J. grandiflorum*, and *J. multiflorum* flowers employing a variety of extraction methods, including solvent extraction and headspace, in addition to seasonal variation, using gas chromatography-mass spectrometry (GC-MS) and multivariate analysis. To analyze the complex dataset, unsupervised multivariate techniques such as Principal Component Analysis (PCA) and Hierarchical Cluster Analysis (HCA) were employed to explore the similarities and differences in volatile metabolites across various species. Additionally, the study evaluated the potential monoamine oxidase A (MAO-A) inhibitory activity of selected *Jasminum* species on the human MAO-A enzyme. Furthermore, the correlation between the volatile chemical profiles of *Jasminum* species and their MAO-A inhibitory properties was examined. Notably, this research represents the first comprehensive investigation into the inhibitory effects of jasmine concrete and absolute on MAO-A enzyme in vitro.

## Results and discussion

### The yield of Jasmine concrete and Jasmine absolute

The percentage production of concrete was computed using flowers, and the absolute was estimated based on jasmine concrete for closed bud and open flower extracts^55^as shown in Table 1. Concrete and absolute percentages were given as:

Percentage of jasmine concrete (on flower basis) = Weight of concrete / Weight of flowers x 100.

Percentage of jasmine absolute (on concrete basis) = Weight of absolute oil / Weight of concrete x 100.

**Table 1:** Concrete and absolute yields (%) from the flowers of four Egyptian Jasmine species collected in August.

Species	Concrete Productivity [%] *	Absolute Productivity [%] #
*J. sambac*	0.52^a^ ± 0.079	61.28
*J. azoricum*	0.81^a^ ± 0.063	47.87
*J. grandiflorum*	0.76^a^ ± 0.038	39.67
*J. multiflorum*	0.96^a^ ± 0.06	45.54

* On flower basis, # on concrete basis.

^a^ The data presented are the average values of three determinations (*n* = 3) ± SD.

The concrete yields from the flowers of four Egyptian jasmine species ranged from 0.52 to 0.96%, with absolute yields ranging from 39.67 to 61.3%. Yields vary depending on the extraction process, solvent, harvest season, geographical origin, soil, climate, and other factors^56,57^. Jasmine concrete and absolute yields from *J. grandiflorum* in India ranged from 0.3 to 0.33% based on the flower weight during the season (end of June until end of August), with an average of 0.31%^58^. Only 0.20% to 0.28% of concrete was produced by Turkish *J. grandiflorum* flowers cultivation^59^. *J. grandiflorum* cultivated in Egypt yielded 0.31% of concrete depending on the floral basis and 54% absolute depending on concrete basis^16^. Concrete yields were from 0.13 to 0.14% for *J. sambac* and 0.27–0.29% for *J. grandiflorum*, with absolute yields of 53–60% from concrete^57^. The concrete of *J. azoricum* yielded 1.1% of volatile components^60^. Furthermore, a methanolic extract of 10 g of *J. multiflorum* flowers yielded 0.15 g, or approximately 1.38% ^62^.

### Chemical profiling of Jasmine species using GC–MS analysis

**Fig. 1:**
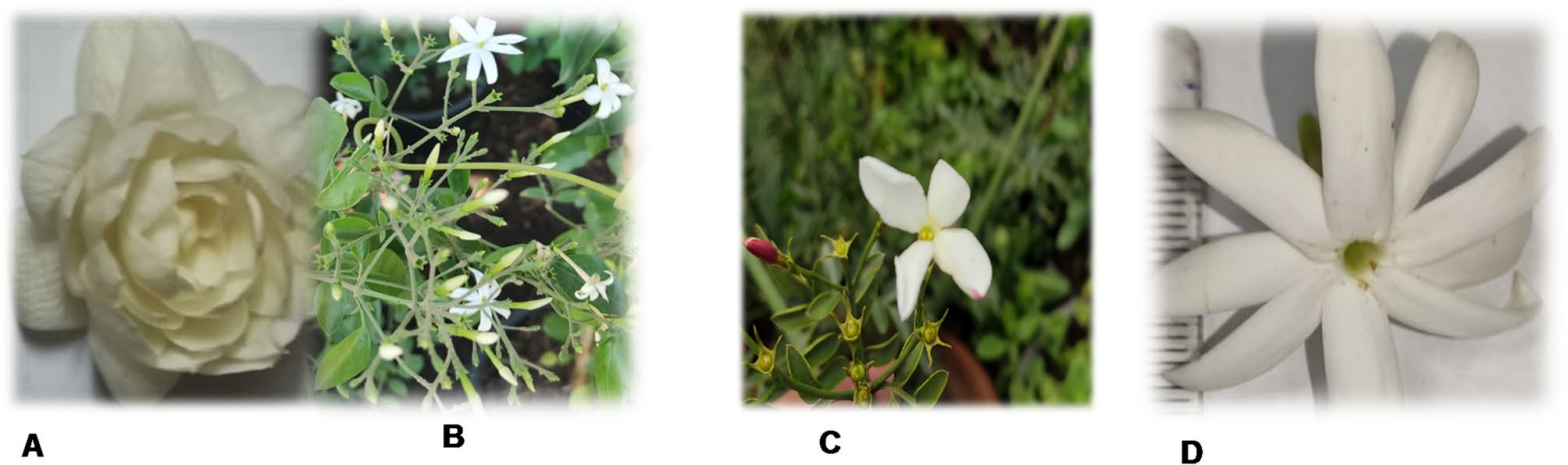
Photographs of the four *Jasminum* species: (**A**) *J. sambac*, (**B**) *J. azoricum*, (**C**) *J. grandiflorum*, and (**D**) *J. multiflorum* flowers collected from Egypt during August 2022.

**Fig. 2:**
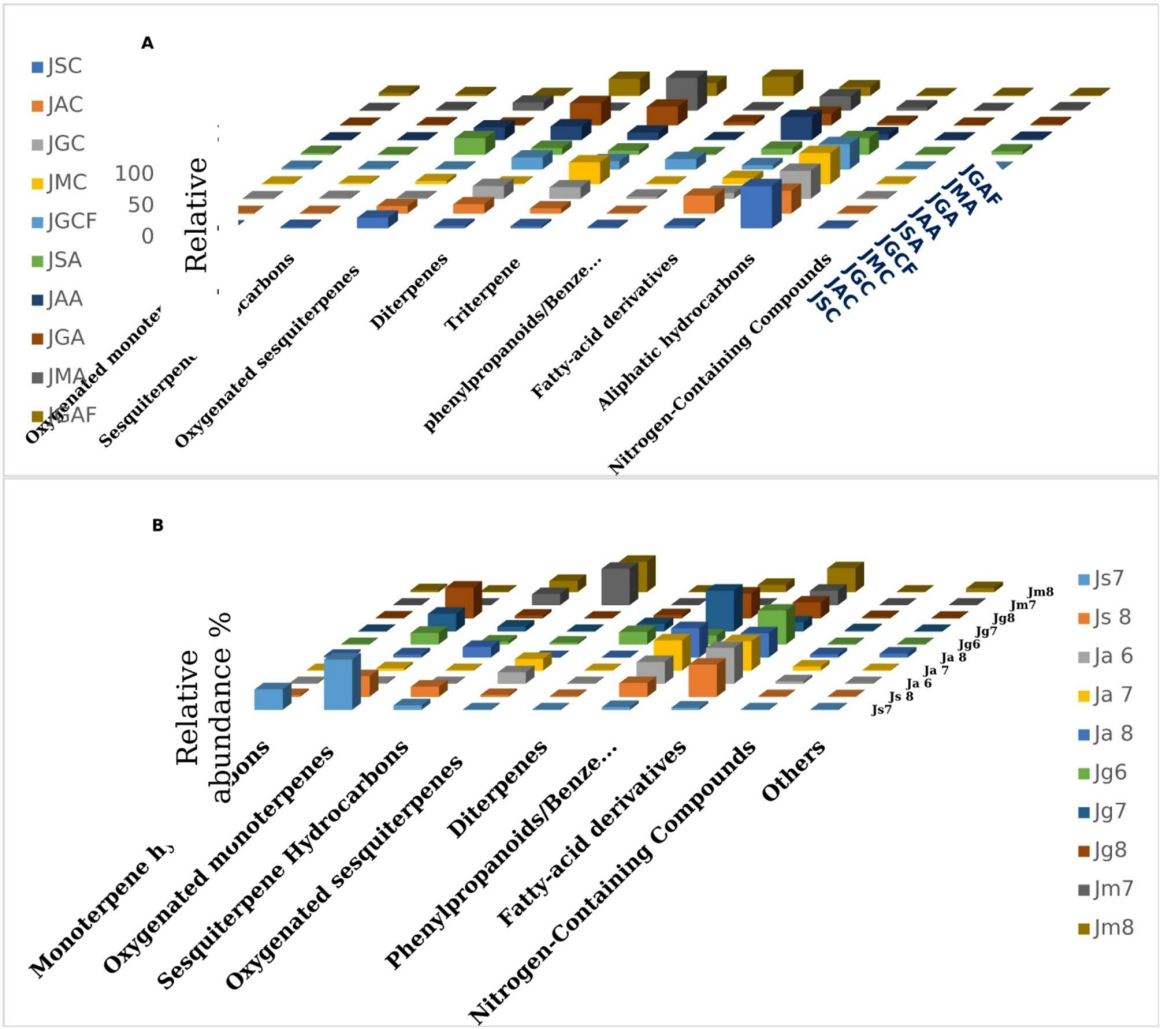
Relative percentile of different classes of volatile compounds identified in four *Jasminum* species (**A**) concrete and absolute. JSC: *J. sambac* Concrete, JAC: *J. azoricum* Concrete, JGC: *J. grandiflorum* Concrete,, JMC: *J. multiflorum* Concrete, JGCF: *J. grandiflorum* Concrete-Factory, JSA: *J. sambac* Absolute, JAA: *J. azoricum* Absolute, JGA: *J. grandiflorum* Absolute, JMA: *J. multiflorum* Absolute, JGAF: *J. grandiflorum* Absolute-Factory. (**B**) headspace volatiles of jasmine flowers collected in June, July and August. Js 7: *J. sambac* flower-July, Js 8: *J. sambac* flower-August, Ja 6: *J. azoricum* flower-June, Ja 7: *J. azoricum* flower-July, Ja 8: *J. azoricum* flower-August, Jg 6: *J. grandiflorum* flower-June, Jg 7: *J. grandiflorum* flower-July, Jg 8: *J. grandiflorum* flower-August, Jm 7: *J. multiflorum* flower-July, Jm 8: *J. multiflorum* flower-August.

Gas chromatography coupled to mass spectrometry (GC-MS) was used to compare the volatile profile of fresh flowers grown in Egypt, as well as concrete and absolute, resulting in the identification of 157 volatile components divided into fourteen classes (Supp. Table S1). Figure 1 shows photographs of the four *Jasminum* species, while Fig. 2 demonstrates the percentiles of major volatile classes of Jasmine flowers concrete and absolute.

#### Monoterpene hydrocarbons

Monoterpene hydrocarbons were one of the most prevalent volatile groups in *J. sambac* flowers collected in July, accounting for 25.41% of the volatile constituents, whereas they were absent from both concrete and absolute samples and present only in the jasmine flower headspace. *cis*-*β*-Ocimene was the primary monoterpene hydrocarbon identified in *J. sambac* flowers collected in July, representing 25.41%. However, in August, the concentration dropped to 1.36%. *cis*-*β*-Ocimene was only found in *J. azoricum* flowers collected in August at 0.86%. Genetic research on single-petal *J. sambac* identified *β*-ocimene as a significant floral volatile, with a relative concentration of 2.23%^62^.

#### Oxygenated monoterpenes

The most abundant volatile classes in *J. sambac* flowers in July and August were oxygenated monoterpenes, which made up 63.06% and 25.52% of the flowers, respectively. Furthermore, during the month of collection, the concentration of oxygenated monoterpenes in *J. grandiflorum* flowers increased from 14.03% in June to 21.71% in July and 38.19% in August. In contrast, *J. multiflorum* had no oxygenated monoterpenes at all, while *J. azoricum* had lesser quantities (0.47–2.26%).

Linalool was the most common oxygenated monoterpene alcohol in the blossom headspace^16^, serving as a major volatile marker in all species except *J. multiflorum* flowers. Linalool dominated the profile of *J. sambac* flowers, accounting for 63.06% in July and 25.41% in August. *J. azoricum* flowers; their concentration was relatively low, accounting for 0.47%, 2.26%, and 2.19% in June, July, and August, respectively. Linalool content of *J. grandiflorum* flowers increased gradually with the seasons, reaching 14.03%, 21.71%, and 38.19% in June, July, and August, respectively. For instance, linalool was absent from *J. multiflorum* flowers. These results were in line with those of Issa et al., who found that the main oxygenated monoterpene alcohol in *J. sambac* and *J. grandiflorum* was *β*-linalool, accounting for 7% in *J. sambac* flowers, 58% in *J. grandiflorum* flowers, and *J. grandiflorum* concretes and absolutes (8% and 11%, respectively)^16^. Linalool is a key component of jasmine’s perfume because of its sweet, woody tone and flowery, fruity scent^63^. Linalool attracts a wide range of pollinators, parasitoids, and herbivores^8^. It had high antibacterial activity against periodontal infections and streptococci. Linalool was utilised in mouthwash and toothpaste for its antibacterial properties^64^.

Bera et al. found similar results, with linalool levels of 12.66% in *J. grandiflorum* flowers, 8.84% in *J. sambac* flowers, and 1.64% in *J. multiflorum* flowers from India^65^. The proportion identifed in *J. grandiflorum* products matched that of concrete and absolute from India, which were around 6% and 8%, respectively^20,66^. Linalool levels are mostly stable at night but increase throughout the day^8^. *Jasminum* blooms, specifically *J. grandiflorum* were collected early in the morning, which explains their high concentration^16^.

In contrast, oxygenated monoterpenes were relatively less prevalent in the concretes and absolutes compared to the flowers′ headspace. Linalool and 2,7-octadiene-1,6-diol,2,6-dimethyl-, were the only oxygenated monoterpenes found in the concretes and absolutes. Linalool was found at 0.73% in *J. sambac* concrete and at greater quantities in purchased *J. grandiflorum* samples, accounting for 1.76% in concrete and 4.35% in absolute. It was not detected in concretes or absolutes from other species that were extracted. Meanwhile, *J. sambac* absolute contained just 2.09% of 2,7-octadiene-1,6-diol, 2,6-dimethyl.

#### Sesquiterpene hydrocarbons

Sesquiterpene hydrocarbons made up 1.24, 0.72, 1.16, and 1.13% of *J. sambac*, *J. grandiflorum*, *J. multiflorum*, and purchased *J. grandiflorum* concrete, respectively. The primary sesquiterpene hydrocarbon identified in concrete and absolute was (*E*, *E*)-*α*-farnesene, which contributed significantly to these values. Additionally, (*E*,* E*)-*α*-farnesene was detected in *J. grandiflorum* absolute at 0.80%, *J. multiflorum* absolute at 1.80%, and the purchased *J. grandiflorum* absolute at 1.99%.

Notably, (*E*, *E*)-*α*-farnesene was found at higher concentrations in the flowers of *Jasminum* species. In *J. sambac* flowers collected in July and August, the predominant sesquiterpene hydrocarbon was (*E*,* E*)-*α*-farnesene, accounting for 5.19% and 10.45%, respectively. Furthermore, *α*-farnesene accounted for 7.53% of the volatile chemicals found in *J. azoricum* flowers harvested in August. (*E*, *E*)-*α*-Farnesene was the only sesquiterpene hydrocarbon identified in *J. grandiflorum* flowers, accounting for 3.08% and 5.34% in flowers collected in June and July, respectively. In addition, sesquiterpene hydrocarbons were the third most abundant class in *J. multiflorum* flowers, with (*E*, *E*)-*α*-farnesene accounting for 13.46% in July and 13.54% in August. The second most prevalent sesquiterpene hydrocarbon observed in *J. azoricum* flowers was *α*-bisabolene, accounting for 5.29%, and it was absent in the remaining species. Yu et al. reported *α*-farnesene as a significant chemical released by jasmine flowers by comparing blooms of *J. sambac* at five developmental phases^67^. Lin et al. analysed the volatile chemicals released from jasmine teas made from *J. sambac* flowers; results showed that *α*-farnesene in premium jasmine teas varied from 14.5% to 16.51% ^14^. (*E*, *E*)-*α*-Farnesene levels were found to be 29.08%, 16.05%, and 5.75% in three Indian species: *J. sambac*, *J. multiflorum*, and *J. grandiflorum*, respectively^65^. It reached 15.9% and 13.1% in *J. sambac* concrete and absolute by HS-SPME from Egypt, respectively^7^. The main sesquiterpene hydrocarbons found in Egyptian *J. grandiflorum*, *J. multiflorum*, and *J. sambac* flowers were (*Z*, *E*)-*α*-farnesene, accounting for 13.08, 12.56, and 15.82%, respectively^16^. Farnesene is responsible for the pleasant, calming scent emitted by *J. sambac*, in addition to the presence of linalool and benzyl acetate as the major volatile components^65^.

#### Oxygenated sesquiterpenes

Oxygenated sesquiterpenes were abundant in jasmine concretes and absolutes. They accounted for 16.11% and 25.69% of the total volatiles in *J. sambac* concrete and absolute, respectively, with farnesol accounting for the majority (9.96% in concrete and 21.26% in absolute). *J. azoricum* concretes and absolutes also contained considerable oxygenated sesquiterpene quantities (11.09% and 19.31%, respectively), with farnesol as the primary compound (9.50% in concrete and 17.04% in absolute). Additional sesquiterpene alcohols were identified in *J. sambac*, including germacrene D-4-ol in *J. sambac* concrete and absolute only at 2.07% and 2.22% of the total volatile content, respectively. Compared to earlier reports, germacrene D-4-ol was identified in *J. sambac* flowers analyzed utilizing five different polarity fibers of SPME at various daytime intervals^68^. In addition, *J. multiflorum* absolute contained 12.13% oxygenated sesquiterpenes, with nerolidol being the main ingredient (11.94%), while the concrete had a lower concentration of these compounds, 3.99%, derived entirely from nerolidol. In *J. sambac* concrete and purchased *J. grandiflorum* absolute, nerolidol was detected at lower concentrations of 0.35% and 0.11%.

Furthermore, *J. azoricum* flowers also contained farnesol, which was found to be 8.42% in June and 10.02% in July. It was reported as a key component in supercritical CO₂ extracts of *J. sambac* flowers from Pakistan at both closed bud and open flower stages, accounting for 8.91% and 8.31%, respectively^55^. Because of its antibacterial qualities, farnesol may be used as a deodorant and is utilized in the perfume industry to enhance the pleasant aromas of floral perfumes^69^. It has been shown to have anticancer and anti-inflammatory properties, as well as to treat asthma due to allergies, gliosis, and edema^70^. Peyrot et al. reported that *J. azoricum* concrete from France comprised 30.1% farnesol^60^. Also, nerolidol was the most predominant oxygenated sesquiterpene in *J. multiflorum* flowers HS in July and August at 45.49% and 37.81%, compared to prior findings of 77, 30, and 12% in Malaysian^61^, Egyptian^16^, and Indian flowers^65^, respectively, using the HS-SPME. Various plants with a flowery scent naturally contain nerolidol (3,7,11-trimethyl-1,6,10-dodecatrien-3-ol), a sesquiterpene alcohol. Cosmetics like shampoos and fragrances, as well as non-cosmetic items like detergents and cleansers, usually include nerolidol^71^.

#### Diterpenes

Diterpenes were most prevalent in *J. grandiflorum*, accounting for 19.67% in concrete and up to 34.78% in absolute, preceded by *J. azoricum* concrete and absolute (14.05% and 21.07%, respectively). Purchased *J. grandiflorum* concretes and absolutes also exhibited high concentrations of diterpenes (18.33% and 26.54%, respectively). Diterpenes were less prevalent in *J. sambac* concrete and absolute (2.86% and 9.98%, respectively). *trans-*Geranylgeraniol was the major diterpene in *J. azoricum*, accounting for 12.28% in concrete and 18.5% in absolute.

In addition, phytol, phytol acetate, isophytol and neophytadiene were detected in *J. grandiflorum* concrete and absolute. In *J. grandiflorum*, the most prevalent diterpene was phytol, which occurred at 5.14% in concrete and 9.69% in absolute, as well as phytol acetate, which accounted for 5.41% and 8.76%, respectively. Similarly, in the purchased *J. grandiflorum*, phytol was identified at 7.22% in the concrete and 8.01% in the absolute, whereas phytol acetate accounted for 3.81% and 6.63%. Furthermore, isophytol was an important diterpene in *J. grandiflorum*, accounting for 5.94% in concrete and 10.52% in absolute. It was similarly plentiful in the purchased *J. grandiflorum*, at 5.03% in concrete and 8.45% in absolute. Neophytadiene was detected in *J. grandiflorum* concrete and absolute at low concentrations, representing 0.17% and 0.20%, respectively. It was also found in the purchased *J. grandiflorum* concrete and absolute, contributing to 0.31% and 0.40%, respectively. *J. grandiflorum* absolute from India displayed trace amounts of neophytadiene (0.1%), determined by GC and in HS-GC ^58^.

The main diterpenes found in *J. grandiflorum* flowers were isophytol, neophytadiene, and (*E*,* E*)-geranyllinalool. Isophytol was the major diterpene in *J. grandiflorum* flowers collected in June, accounting for 7.99%; it was also detected in July at 4.72% and in August at 0.33%. Neophytadiene was detected at 4.03% in June, 2.26% in July, and 2.96% in August. It was previously reported in *J. grandiflorum* from China (0.23%) extracted using hydro-distillation^72^. The concentration of (*E*,* E*)-geranyllinalool peaked in June at 1.62%, then fell to 0.64% in July. Furthermore, in August, this compound was detected in 0.38% in *J. sambac* flowers.

#### Triterpenes

Triterpenes were absent in the HS volatiles of *J. sambac*, *J. azoricum*, *J. grandiflorum*, and *J. multiflorum* flowers. Meanwhile, triterpenes were detected mainly in *J. multiflorum* concrete and absolute at 33.99% and 51.42%, represented mainly by 2,3-epoxysqualene (33.17% and 49.4%) and squalene, though at trace amounts. Triterpenes were the second most common volatile class in *J. multiflorum* concrete after aliphatic hydrocarbons, although triterpenes were the major class detected in *J. multiflorum* absolute (Fig. 2A). Besides, *J. grandiflorum* concrete and absolute displayed a higher percentage of 2,3-epoxy squalene, at 12.03 and 20.98% vs. 8.55 and 13.25% in purchased *J. grandiflorum* concrete and absolute. 2,3-Epoxysqualene was found in lower concentrations in *J. sambac* concrete and absolute (1.12 and 1.32%) and *J. azoricum* concrete and absolute (3.27 and 4.96%), respectively. Egyptian absolutes of all four jasmine species, as well as the purchased products, displayed more squalene and 2,3-epoxysqualene, however lower amounts were detected in Chinese and Indian *J. sambac* flower absolutes^17^. 2,3-Epoxy squalene accounted for 20.98% in *J. grandiflorum* absolute in the current study; however, lower concentrations were reported in *J. grandiflorum* absolute from India, Egypt, and Morocco^73^.

Other triterpenes identified in all specimens include squalene. Squalene levels were much lower in *J. multiflorum* concrete and absolute (0.82% and 1.18%, respectively), as well as *J. sambac* concrete (1.41%), while *J. sambac* absolute displayed a higher squalene concentration of 4.44%. On the other hand, squalene was detected in a higher proportion in *J. grandiflorum* concrete and absolute, at 5.62% and 8.13% vs. 3.86% and 6.52% in the purchased *J. grandiflorum* concrete and absolute. In *J. azoricum*, concrete and absolute, squalene was present at 4.16% and 5.66%, respectively. The level of squalene in the current study was consistent with the analysis of twenty *J. grandiflorum* commercial flower absolutes from various sources, including Egyptian and Moroccan absolutes, as well as market Indian flower absolutes^73^. Two commercial absolute samples from India, one for *J. grandiflorum* and one for *J. sambac*, revealed levels of squalene at 4.6 and 1.2% and 2,3-epoxy squalene at 11.7 and 0.6%, respectively^57^. Squalene and 2,3-epoxy squalene were not detected in prior SPME examination of Egyptian *J. sambac*, *J. grandiflorum* and *J. multiflorum* blossoms’ headspace volatiles, concrete, and absolute^16,55^.

#### Phenylpropanoids/Benzenoids

Phenylpropanoids/benzenoids were the third most prevalent volatile class in the purchased *J. grandiflorum* concrete, accounting for 15.76%, and the most abundant class in the purchased *J. grandiflorum* absolute, accounting for 29.79%. Benzyl acetate was the main volatile constituent in purchased *J. grandiflorum* concrete and absolute at 9.39% and 17.93%, respectively, followed by benzyl benzoate, accounting for 5.59% and 9.33%, respectively. Benzyl benzoate was the major volatile constituent in *J. grandiflorum* absolute at 3.04%. In fragrance, benzyl benzoate is utilized as a fixative^74^. Additionally, benzyl benzoate has antibacterial activity against gram-negative bacteria^75^and is helpful for reducing hypertension^76^. In a single sample, it barely reached 0.5% and 0.6% in the concrete and absolute of *J. grandiflorum* from India ^67^ and 21% in the absolute of a different sample from India^20^. Benzyl benzoate was the main ester in *J. grandiflorum* concrete and absolute from Egypt, accounting for ca. 2–5% ^16^.

The phenylpropanoids and benzenoids in the *Jasminum* flowers under study showed significant seasonal fluctuation. It were the most common volatile class in *J. azoricum* flowers collected in July and August, as well as *J. grandiflorum* flowers collected in July, accounting for 37.67%, 36.51%, and 50.89%, respectively. It was the second most prevalent class in *J. sambac* flowers collected in August, *J. azoricum* flowers obtained in June, and *J. grandiflorum* flowers collected in August, accounting for 16.73%, 26.76%, and 30.79%, respectively. Overall, the findings show that benzenoids and phenylpropanoids were more prevalent in flower headspace volatiles than in absolutes and concretes.

Benzyl acetate was the primary benzenoid ester, which exhibits a distinct jasmine aroma and exhibited notable seasonal variation among the investigated species. It was particularly prevalent in *J. grandiflorum* flowers, peaking at 47.68% in July and then falling to 23.32% in August and 4.29% in June. Notably, this significantly elevated percentage in July helped make benzyl acetate the main component of *J. grandiflorum’s* flowers phenylpropanoid and benzenoid class. Benzyl acetate was found in *J. sambac* flowers only in August at 8.95%, although its levels varied considerably in *J. azoricum* flowers (8.16% in June, 0.63% in July, and 10.57% in August). Herein, the three jasmine species were found to contain benzyl acetate, but *J. multiflorum* flowers did not. According to Issa et al. ^16^, benzyl acetate was absent in *J. multiflorum* flowers, whereas it accounted for 12.87% of *J. multiflorum* flowers from India. Differences were observed in the benzyl acetate content of jasmine varieties, even among the same species^16^.

Benzyl alcohol, another key benzenoid that contributes to jasmine’s flowery, sweet, and somewhat rose scent^77^, exhibited considerable seasonal variation in concentrations. It was significantly abundant in *J. azoricum* flowers, reaching 11.02% in June, peaking at 25.39% in July, and somewhat declining to 13.43% in August, making it one of the species’ most important contributors to this category. In comparison, *J. grandiflorum* flowers had very low levels (3.54% in June, 0.82% in July, and 1.04% in August). In *J. sambac*, benzyl alcohol was detected at trace levels (0.96%) in June but grew to 4.76% in August, whereas it was not found in *J. multiflorum*.

The headspace of *J. grandiflorum* and *J. multiflorum* flowers contained benzyl benzoate, but *J. sambac* and *J. azoricum* did not. *J. grandiflorum* showed reasonably steady but low levels during the three sample months, accounting for 1.83% in June, 1.25% in July, and 1.76% in August. In contrast, *J. multiflorum* only displayed benzyl benzoate at 1.37% in August, with no detectable concentrations in July. Bera et al. ^66^ found the presence of benzyl benzoate in the headspace volatiles of *J. multiflorum* (0.86%) and *J. grandiflorum* (2.54%), which is consistent with our findings and further supports its function as a distinctive benzenoid characteristic of these species.

Another benzenoid ester found in the studied jasmine blossoms was *cis*-3-hexenyl benzoate, which varied in concentration between species and collecting months. It was detected in *J. sambac* flowers only in August at 0.89% and in *J. azoricum* flowers in August at 0.59%. *cis*-3-Hexenyl benzoate was more consistently present in *J. grandiflorum*, with concentrations of 0.98%, 1.14%, and 1.10% in June, July, and August. Remarkably, in August, *J. multiflorum* had the greatest percentage of *cis*-3-hexenyl benzoate (1.69%). These results are consistent with those found by Bera et al. ^66^, who found that *J. sambac* had *cis*-3-hexenyl benzoate at 1.46%, *J. multiflorum* at 3.01%, and *J. grandiflorum* at 0.70%.

Some benzenoids esters are exclusive to specific specimens, such as methyl anthranilate, found only in *J. sambac*. Methyl anthranilate reached 1.12% and 0.42% in the *J. sambac* flowers and concrete. It was the only ester that has been found to include nitrogen, which has an orange blossom flavour^7^. Edris et al. stated that methyl anthranilate was present at 5% and 4.7% in Egyptian concrete HS and the absolute of *J. sambac* flowers, respectively^7^.

Eugenol (2.63%) and *p*-cresol (0.94%) were two more phenylpropanoids/benzenoids that were present in *J. grandiflorum* flowers. *p*-Cresol was also found in *J. azoricum*, accounting for 2.32% in June, 3.42% in July, and 2.55% in August. Neither eugenol nor *p*-cresol was detected in *J. sambac* and *J. multiflorum* flowers. Eugenol was absent in Egyptian *J. sambac* concrete and absolute in a study conducted by Edris et al.. ^7^, which was consistent with the current study. Additionally, eugenol was absent in the blooms of *J. sambac*, *J. multiflorum*, and *J. grandiflorum* grown in India or Malaysia^65,68^. Eugenol was reported as a major component in the headspace volatiles of Egyptian *J. sambac* flower, accounting for 9.3% ^16^. In contrast, it was found in lower amounts in *J. multiflorum* and *J. grandiflorum* flowers at 0.15% and 1.03%, respectively^16^. Jirovetz et al. found that 2.5% of the absolute of *J. grandiflorum* from India was eugenol^20^.

Benzeneacetaldehyde was the major phenylpropanoid/benzenoid (aldehyde) found in *J. multiflorum* blooms (2.93%), and it was absent from other species. It accounted for 2% in *J. multiflorum* flowers from Egypt^16^. Benzaldehyde was detected in trace amounts in *J. sambac* flowers at 0.09% collected in August and identified in *J. sambac* flowers from Egypt at 1.19% ^16^. While *J. multiflorum* flowers in Malaysia lacked aldehydes, *J. sambac* flowers showed high levels of benzaldehyde and 2-phenylacetaldehyde (17.92% and 10.24%, respectively)^61^. In addition, benzaldehyde was found at 0.1% in *J. sambac* concrete from Egypt, but it was absent in the absolute^7^. It was found in trace concentrations in one of the three *J. sambac* concrete samples that were obtained from China^78^.

#### Fatty acid derivatives

Fatty acid derivatives were most abundant in *J. azoricum* concrete and absolute at 27.66% and 36.02%, respectively. In *J. sambac*, they accounted for 3.61% in concrete and 8.95% in absolute. *J. grandiflorum* demonstrated moderate levels with 8.69% in concrete and 17.46% in absolute, whereas *J. multiflorum* revealed 8.99% in concrete and the greatest concentration in the absolute, 22.03%. In contrast, the purchased *J. grandiflorum* concrete and absolute products revealed a lower percentage of fatty acid derivatives compared to the laboratory-extracted *J. grandiflorum*, accounting for 6.06% and 13.13%, respectively, as illustrated in the fatty acid derivative concentrations (Fig. 2A). The major fatty acid derivative ester detected in *J. azoricum* was found to be 9,12-octadecadienoic acid (*Z*,* Z*)-, methyl ester, also known as methyl linoleate, accounting for 10.36% of the concrete and 14.99% of the absolute. Methyl linoleate was present at 5.8% in *J. azoricum* concrete from France^60^.

Some fatty acid derivatives were exclusive to *J. grandiflorum* concrete and absolute, including methyl jasmonate and its isomer methyl epijasmonate. In the present study, methyl epijasmonate concentration in *J. grandiflorum* (0.52 and 0.9% for concrete and absolute, respectively) was almost identical to that reported for *J. grandiflorum* absolute and concrete from Egyptian origin^16^. *cis*-Methyl jasmonate is the primary cause of the floral, flowery, and fruity odours in *J. grandiflorum* flowers^20^. It has been demonstrated that the bioactive component methyl jasmonate (MJ), which was isolated from *J. grandiflorum*, exhibited antidepressant properties^79^.

Additionally, another jasmonate-related molecule, *cis*-jasmone, was identified in *J. grandiflorum* concrete and absolute at 0.33 and 0.86%, respectively, and in the purchased *J. grandiflorum* concrete and absolute at 1.05 and 2.57%, respectively. *J. sambac* and *J. azoricum* did not contain *cis*-jasmone in either concrete or absolute form. In contrast, *J. multiflorum* absolute had just traces (0.39%). Reports showed that it was absent in *J. sambac* concrete and absolute growing in Egypt collected in July^7^. Furthermore, it was reported at 1.9% in *J. grandiflorum* absolute from India^20^. Jasmone was found in low amounts in two of three Chinese *J. sambac* concretes^78^. Jasmone is a fragrance ingredient advised to be used in perfume compositions, particularly for bases, specialities, and synthetic jasmine manufacturing because of its potent jasmine-like scent and faint fruity-warm spicy aroma^80^. *cis*-Jasmone is a component found in several fragrances. It can be found in non-cosmetic products such as household cleaners and detergents, as well as perfumes used in decorative cosmetics, fine scents, shampoos, bath soaps, and other toiletries^81^. In addition, jasmine lactone was identified in the purchased *J. grandiflorum* absolute at 0.33%. Jasmine lactone is responsible for the intense, sweet jasmine aroma and fruity odor^82^. Jasmine lactone accounted for 0.27–0.81% in *J. grandiflorum* products (absolute and concrete)^16^. Jasmolactone was also found in Malaysian *J. multiflorum* extracted with methanol at 12%, but it was absent in *J. sambac*^61^. However, *δ*-jasmine lactone was shown to be present in 1.1% of the Indian *J. grandiflorum* absolute^20^. Moreover, methyl palmitate was found in the concretes at 0.29%, 3.53%, 1.61%, and 0.71% in *J. sambac*, *J. azoricum*, *J. grandiflorum*, *and J. multiflorum*, respectively, whereas 1.0% was found in the purchased *J. grandiflorum* concrete. Methyl palmitate was detected in the absolutes in *J. azoricum*, *J. grandiflorum*, and *J. multiflorum* at 5.47%, 2.44%, and 1.92%, respectively, while it was present in the factory *J. grandiflorum* absolute at 2.14%. By contrast, Edris, Chizzola et al. found that methyl palmitate made up 0.02 and 0.6% of *J. sambac* concrete HS-SPME and absolute from Egypt, respectively^7^, whereas the *J. grandiflorum* absolute sample from India contained 1.4% ^20^, which was consistent with our findings.

While fatty acid derivatives differed significantly between concretes and absolutes, they were also the most abundant volatile groups in *J. sambac* flowers collected in August, accounting for 40% of the volatile contents. In *J. azoricum* flowers collected between June and August, fatty acid derivatives accounted for 44.66%, 36.59%, and 30.17%, respectively. Fatty acid derivatives in *J. grandiflorum* flowers showed significant seasonal variation, with the highest concentration in June (42.23%), followed by a severe fall in July (10.76%) and a minor increase in August (19.35%). Similarly, *J. multiflorum* followed a seasonal variation, accounting for 17.77% in July and increasing to 29.66% in August. The primary fatty acid ester found in the HS volatiles of *J. sambac* flowers was *cis*-3-hexenyl-1-acetate (33.09%). Noteworthy, *cis*-3-hexenyl-1-acetate was absent in *J. sambac* concrete or absolute. This agrees with previous studies, which showed that *cis*-3-hexenyl acetate is more abundant in the HS of flowers than the essential oil, concrete, or absolute due to its high volatility^68^. (*Z*)-3-Hexenyl-1-acetate was also identified in HS of *J. sambac* flowers from Egypt, representing 18.11% ^16^, and 10.6% in *J. sambac* absolute from India, analyzed by vacuum headspace^57^. Besides, Bera et al. reported that Indian *J. sambac* and *J. multiflorum* flowers contained 7.5% and 1.8% of (*Z*)-3-hexenyl acetate, respectively^65^.

Additionally, *cis*-jasmone was the most abundant fatty acid derivative in *J. multiflorum* flowers (11.62%), as well as in *J. grandiflorum* (10.29%), whereas it was absent in *J. sambac* and *J. azoricum* flowers. Furthermore, employing five distinct polarity SPME fibers, *cis*-jasmone was absent in Indian *J. sambac* flowers, which agrees with the study herein^68^. *cis*-Jasmone was found only in negligible amounts in *J. sambac* flowers but reached 9.0% and 0.8% in *J. multiflorum* and *J. grandiflorum* flowers grown in India, respectively^65^. It was reported to be present at 15.31% in *J. multiflorum* examined by SPME and 4.06% in *J. multiflorum* extracted using methanol, but not in Malaysian *J. sambac* flowers^61^. *cis*-Jasmone was found at 8.90% in *J. grandiflorum*, 26.61% in *J. multiflorum*, and 12.23% in *J. sambac* flowers from Egypt^16^. Moreover, jasmine lactone was identified in the flowers of two jasmine species, *J. grandiflorum* and *J. multiflorum*, at 1.89% and 0.59%, respectively. These findings align with earlier reports where jasmine lactone accounted for 1.76% in Egyptian *J. multiflorum* flowers analyzed by SPME^16^.

Furthermore, methyl palmitate (a fatty acid methyl ester) was responsible for 15.76%, 3.56%, and 1.36% of the total fragrance released by *J. azoricum*, *J. multiflorum*, and *J. grandiflorum* flowers, respectively, whereas it only accounted for 0.05% in *J. sambac* flowers collected in August. Methyl palmitate showed a distinct seasonal fluctuation in *J. azoricum* flowers, peaking in August at 15.76%, whereas July and June saw 10.67% and 12.5%, respectively. Methyl palmitate and 9,12-octadecadien-1-ol were the most prevalent fatty acid derivatives found in this species, highlighting their significant contribution to the fatty acid composition of *J. azoricum* flowers.

(*E*)-2-Hexenal, a fatty acid derivative (aldehyde), was most commonly found in *J. grandiflorum* flowers, with significant amounts in June (27.5%) and a lesser proportion in August (4.28%). It was also prevalent in *J. multiflorum* flowers, reaching 7.9% in August and 2.59% in July, but it was only found in trace amounts in *J. sambac* flowers (0.35%). *J. grandiflorum*, *J. multiflorum*, and *J. sambac* flowers from Egypt displayed 2.74%, 0.68%, and 0.55% of 2-hexenal, respectively^16^.

#### Aliphatic hydrocarbons

This class was absent in the HS volatiles of *J. sambac*, *J. azoricum*,* J. grandiflorum*, and *J. multiflorum* flowers. Meanwhile, aliphatic hydrocarbons (alkanes) accounted for approximately 66.3% of *J. sambac* concrete and were present at lower levels in *J. sambac* absolute at 25.78%. Aliphatic hydrocarbons accounted for about 48.81% of *J. multiflorum*, 43.91% of *J. grandiflorum*, 35.34% of *J. azoricum*, and 39.65% of the purchased *J. grandiflorum* concrete (Fig. 2A). *J. azoricum*, *J. grandiflorum*, *J. multiflorum*, and purchased *J. grandiflorum* absolutes displayed lower concentrations of this class, accounting for 8.54%, 2.98%, 4.43%, and 0.2%, respectively.

The major aliphatic hydrocarbons in *J. sambac* concrete were hentriacontane, nonacosane, 9-tricosene, tritriacontane, and pentacosane at ca. 22.67%, 11.88%, 6.11%, 3.96%, and 3.92%, respectively. Aliphatic hydrocarbons were the most prevalent volatile class in *J. sambac* absolute, as illustrated in Fig. 2A, with (*Z*)-9-tricosene being the predominant component, followed by pentacosane and tricosane at 13.77%, 4.01%, and 3.91%, respectively. (*Z*)-9-Tricosene has been detected only in *J. sambac* and was absent in other *Jasminum* species. It was reported in seven *J. sambac* absolutes from India, bud absolute, and commercially available *J. sambac* flower absolutes from India and China at 3.5–13% studied between 2005 and 2013 ^17^.

Hentriacontane and nonacosane were found in *J. azoricum* (8.66% and 8.48%), *J. grandiflorum* (11.75% and 14.52%), *J. multiflorum* (12.32% and 19%), and *J. grandiflorum* factory concrete (9.98% and 12.55%). Nonacosane was not detected in *J. sambac* absolute or purchased *J. grandiflorum* absolute but was found in trace amounts in *J. azoricum*, *J. grandiflorum*, and *J. multiflorum* absolute. Some essential oils, including tetracosane^83,84^, showed antiviral properties.

#### Nitrogen-Containing compounds

Nitrogenous compounds were found in trace amounts throughout the examined *Jasminum* species. They were found in *J. sambac* flowers collected in August at 0.03%. In *J. azoricum*, they followed a seasonal trend, rising from 2.25% in June to 4.5% in July before falling slightly to 3.55% in August. In *J. grandiflorum*, they were reported at 0.82% in July and 0.98% in August, represented by benzyl nitrile, was only identified in *J. azoricum* flowers, indicating strong seasonal change. It peaked in July at 4.5%, followed by 3.55% in August and 2.25% in June, which was absent in the other species. Benzyl nitrile was previously detected in Egyptian *J. sambac* concrete and absolute^7^. In addition, the high temperature at the GC injector port may cause phenylacetaldoxime to dehydrate, resulting in the formation of this artifact^7^. Benzyl nitrile was found in the headspace volatiles of *J. sambac* flowers from India, collected at different times of the day, utilizing five distinct SPME whole polarity fibers^68^. It was identified in the HS-SPME of *J. multiflorum* flowers growing in Egypt at around 1.2% ^16^.

This class was less abundant in *J. sambac*, and *J. grandiflorum* flowers being represented only by indole. It was identified at 0.03% in August in *J. sambac* flowers and at 0.82% in July and 0.98% in August in *J. grandiflorum* flowers. Nitrogen-containing floral smell molecules are produced through amino acid metabolism. Indole is created by the direct breakdown of the tryptophan precursor and indole-3-glycerol phosphate^85^. Indole was found at 1.83% in the volatile components of fresh *J. sambac* flowers and Chinese Jasmine Tea^86^. It was absent in Malaysian *J. sambac* flowers and was only present at 0.04% in *J. multiflorum* analyzed by HS-SPME^61^. Previous research showed that *J. multiflorum* and *J. sambac* flowers cultivated in Egypt and analyzed by HS-SPME contained indole at 0.65% and 0.15%, respectively. Besides, the concrete and absolute corresponded to the indole-rich type^16^. Indole was shown to be a significant volatile ingredient in *J. multiflorum* flowers from India, accounting for 10.3%. It was also present in *J. sambac* and *J. grandiflorum*, with concentrations of 1.94 and 6.49%, respectively^65^. Indole was reported as one of the main compounds in the concrete and absolute of Egyptian *J. sambac*, accounting for 13.1 and 13.4%, respectively^7^. *J. sambac* flowers from India were found to contain indole using (HS-SPME) with five different polarity fibers to analyze the flowers at different times of the day. Using polyacrylate (PA), indole (15.7%) was identified in larger proportions compared with DVB/Carboxen/PDMS, which was the lowest quantity (0.3%) among other fibers^68^. The percentage of indole in jasmine affects its quality and modulates the floral odors of other substances, according to several publications^87^. Our samples showed a low indole fraction, indicating that this species is indole poor, similar to headspace SPME investigations on *J. sambac* from India^88^, ^69^.

**Table 2:** HS-GC-MS analysis of volatile constituents in the headspace fragrance of *Jasminum sambac* (Js*)*, *Jasminum azoricum* (Ja), *Jasminum grandiflorum* (Jg), and *Jasminum multiflorum* (Jm) flowers collected in June (6), July (7), and August (8).

Peak	RT	Compound Name	RI	RI(REF)	Js 7	Js 8	Ja 6	Ja 7	Ja 8	Jg 6	Jg 7	Jg 8	Jm 7	Jm 8
1	4.329	Butyl acetate	806	804	-	0.2	-	-	-	-	-	0.07	-	-
2	4.341	*n*-Octane	807	801	-	-	-	-	4.35	-	-	-	-	-
3	4.424	4,4-Dimethyl-3-oxopentanenitrile	810	817	-	-	-	-	-	-	-	-	-	0.87
4	4.909	(*E*)-2-Hexenal	827	827	-	0.35	-	-	-	27.5	-	4.28	2.59	7.9
5	5.178	(*E*)-3-Hexenol	837	836	2.29	0.14	-	-	-	0.48	-	0.12	-	1.7
6	5.247	(*Z*)-Hex-3-en-1-ol	840	840	-	2.15	-	-	-	-	-	-	-	-
7	5.381	3-Methylbutanal oxime	845	858	-	-	-	-	-	-	-	-	-	0.38
8	5.446	(1*Z*)-2-Methylbutanal oxime	847	-	-	-	-	-	-	-	-	-	-	1.97
9	5.535	(*E*)-2-Hexen-1-ol	850	850	-	0.8	-	-	-	-	-	-	-	-
10	5.633	1-Hexanol	854	854	-	0.36	-	-	-	-	-	-	-	-
11	5.646	(1*E*)-2-Methylbutanal oxime	854	-	-	-	-	-	-	-	-	-	-	1.17
12	5.808	1-Butanol, 3-methyl-, acetate	860	860	-	0.03	-	-	-	-	-	-	-	-
13	6.963	(*Z*)-2-Pentenyl acetate	902	897	-	0.25	-	-	-	-	-	-	-	-
14	7.61	Benzaldehyde	926	926	-	0.09	-	-	-	-	-	-	-	-
15	9.316	*β*-Pinene	988	988	-	0.07	-	-	-	-	-	-	-	-
16	9.466	*cis*-3-Hexenyl-1-acetate	993	990	-	33.09	-	-	-	-	-	0.55	-	-
17	9.723	*p*-Methylanisole	1002	1003	-	-	-	-	0.67	-	-	-	-	-
18	9.729	*trans*-2-Hexenyl acetate	1003	997	-	2.39	-	-	-	-	-	-	-	-
19	9.956	Benzyl alcohol	1010	1008	0.96	4.76	11.02	25.39	13.43	3.54	0.82	1.04	-	-
20	9.974	Benzeneacetaldehyde	1010	1009	-	-	-	-	-	-	-	-	-	2.93
21	10.667	*trans*-*β*-Ocimene	1033	1032	-	0.02	-	-	-	-	-	0.26	-	-
22	11.004	*cis*-*β*-Ocimene	1044	1043	25.41	1.36	-	-	0.86	-	-	-	-	-
23	11.457	*p*-Cresol	1058	1057	-	-	2.32	3.42	2.55	-	-	0.94	-	-
24	11.608	Dihydro myrcenol	1063	1062	-	-	-	-	1.18	-	-	-	-	-
25	11.854	Methyl benzoate	1071	1070	-	-	-	-	-	-	-	-	-	0.19
26	12.33	Phenylethyl Alcohol	1086	1086	-	-	2.8	4.88	1.03	-	-	-	-	-
27	12.441	Linalool	1090	1090	63.06	25.41	0.47	2.26	2.19	14.03	21.71	38.19	-	-
28	12.529	Benzyl nitrile	1093	1094	-	-	2.25	4.5	3.55	-	-	-	-	-
29	13.114	(*E*)-4,8-Dimethyl-1,3,7-nonatriene	1111	1104	-	0.12	-	-	0.58	-	-	-	-	1.76
30	13.896	Benzyl acetate	1136	1137	-	8.95	8.16	0.63	10.57	4.29	47.68	23.32	-	-
31	14.908	Methyl salicylate	1169	1169	-	0.31	2.46	2.83	3.67	-	-	-	-	0.41
32	15.049	(*Z*)-Butanoic acid, 3-hexenyl ester	1174	1173	-	0.13	-	-	-	-	-	0.28	-	-
33	15.274	(2*E*)-2-Hexenyl butyrate	1181	1177	-	0.02	-	-	-	-	-	-	-	-
34	16.682	*β*-Phenethyl acetate	1228	1229	-	-	-	-	0.83	-	-	-	-	-
35	17.009	*cis*-Geraniol	1240	1239	-	0.11	-	-	-	-	-	-	-	-
36	17.232	Ethyl salicylate	1248	1249	-	0.1	-	0.52	2.07	-	-	-	-	-
37	17.442	Indole	1255	1255	-	0.03	-	-	-	-	0.82	0.98	-	-
38	17.98	Cinnamyl alcohol	1274	1270	-	-	-	-	1.1	-	-	-	-	-
39	18.935	Methyl anthranilate	1307	1311	1.98	1.12	-	-	-	-	-	-	-	-
40	19.612	Eugenol	1331	1331	-	-	-	-	-	-	-	2.63	-	-
41	20.599	Neryl acetate	1365	1365	-	0.18	-	-	-	-	-	-	-	-
42	20.633	(*Z*)-Jasmone	1367	1367	-	-	-	-	-	7.47	8.27	10.29	15.18	11.62
43	21.896	2-Methylbutyl benzoate	1412	1409	-	-	-	-	-	-	-	-	-	0.76
44	22.711	Jasmine lactone	1444	1442	-	-	-	-	-	0.92	0.73	1.89	-	0.59
45	22.884	*α*-Bisabolene	1451	1443	-	-	-	-	5.29	-	-	-	-	-
46	22.907	(*E*)-*β*-Farnesene	1451	1457	-	0.31	-	-	-	-	-	-	-	-
47	23.521	Germacrene D	1475	1475	-	0.32	-	-	-	-	-	-	-	-
48	23.919	Bicyclogermacrene	1491	1491	-	0.13	-	-	-	-	-	-	-	-
49	24.011	*α*-Muurolene	1495	1495	-	0.03	-	-	-	-	-	-	-	-
50	24.154	(*E*,*E*)-*α*-Farnesene	1500	1500	5.19	10.45	-	-	7.53	3.08	5.34	2.73	13.46	13.54
51	24.325	*γ*-Cadinene	1507	1507	-	0.36	-	-	-	-	-	-	-	-
52	24.502	*α*-Cuprenene	1514	1514	-	0.47	-	-	-	-	-	-	-	-
53	24.56	(+)-*δ*-Cadinene	1516	1516	-	0.16	-	-	-	-	-	-	-	-
54	24.927	*α*-Cadinene	1530	1534	-	0.03	-	-	-	-	-	-	-	-
55	25.253	(*Z*)-3-Hexen-1-yl-benzoate	1543	1542	-	0.89	-	-	0.59	0.98	1.14	1.1	-	1.69
56	25.406	(*E*)-Nerolidol	1549	1549	-	0.53	-	-	-	-	-	-	45.49	37.81
57	25.495	Hexyl benzoate	1553	1551	-	0.14	-	-	-	-	-	-	-	0.76
58	25.603	(*E*)-2-Hexenyl benzoate	1557	1556	-	0.19	-	-	-	-	-	-	-	-
59	25.938	(3*E*,7*E*)-4,8,12-Trimethyltrideca-1,3,7,11-tetraene	1570	1566	-	0.03	-	-	-	-	-	-	-	-
60	27.22	*τ*-Cadinol	1622	1622	-	0.28	-	-	-	-	-	-	-	-
61	27.513	*α*-Cadinol	1635	1634	-	0.19	-	-	-	-	-	-	-	-
62	29.033	(*Z*,*E*)-Farnesol	1701	1699	-	-	8.42	10.02	-	-	-	-	-	-
63	29.452	Benzyl Benzoate	1719	1719	-	-	-	-	-	1.83	1.25	1.76	-	1.37
64	31.422	Benzoic acid, 2-phenylethyl ester	1805	1829	-	-	-	-	-	-	-	-	-	0.55
65	31.562	all-*trans*-Farnesyl acetate	1812	1814	-	0.35	-	-	-	-	-	-	-	-
66	31.649	Nerolidyl acetate	1816	1816	-	0.34	5.39	4.17	-	-	-	-	-	-
67	31.911	Hexahydrofarnesyl acetone	1829	1830	-	-	-	-	-	1.68	-	-	-	-
68	32.115	Neophytadiene	1839	1839	-	-	-	-	-	4.03	2.26	2.96	-	-
69	33.575	Methyl palmitate	1908	1908	-	0.05	12.5	10.67	15.76	3.33	1.4	1.36	-	3.56
70	34.191	Isophytol	1938	1938	-	-	-	-	-	7.99	4.72	0.33	-	-
71	35.594	(*E*,*E*)-Geranyllinalool	2006	2008	-	0.38	-	-	-	1.62	0.64	-	-	-
72	36.773	9,12-Octadecadien-1-ol, (*Z*,*Z*)-	2071	2069	-	-	24.1	20.48	11.79	-	-	-	-	1.59
73	36.837	Methyl linolenate	2074	2073	-	0.04	7.68	5.44	2.62	1.8	0.36	0.51	-	1.62
74	36.969	Methyl elaidate	2081	2084	-	-	-	-	-	0.73	-	-	-	0
75	37.511	Methyl stearate	2111	2111	-	-	0.38	-	-	-	-	-	-	1.08
76	39.236	2-Hexadecen-1-ol, 3,7,11,15-tetramethyl-, acetate, [R-[R*,R*-(*E*)]]-	2206	2232.3	-	-	-	-	-	1.44	0.9	-	-	-
**Monoterpene hydrocarbons** **Oxygenated monoterpenes** **Sesquiterpene hydrocarbons** **Oxygenated sesquiterpenes** **Diterpenes** **Phenylpropanoids/Benzenoids** **Fatty-acid derivatives** **Nitrogen-Containing Compounds** **Others** **Total identified (%)**					**25.41**	**1.57**	**0**	**0**	**1.44**	**0**	**0**	**0.26**	**0**	**1.76**
					**63.06**	**25.52**	**0.47**	**2.26**	**3.37**	**14.03**	**21.71**	**38.19**	**0**	**0**
					**5.19**	**12.29**	**0**	**0**	**12.82**	**3.08**	**5.34**	**2.73**	**13.46**	**13.54**
					**0**	**1.69**	**13.81**	**14.19**	**0**	**1.68**	**0**	**0**	**45.49**	**37.81**
					**0**	**0.38**	**0**	**0**	**0**	**15.08**	**8.52**	**3.29**	**0**	**0**
					**2.94**	**16.73**	**26.76**	**37.67**	**36.51**	**10.64**	**50.89**	**30.79**	**0**	**8.66**
					**2.29**	**40**	**44.66**	**36.59**	**30.17**	**42.23**	**10.76**	**19.35**	**17.77**	**29.66**
					**0**	**0.03**	**2.25**	**4.5**	**3.55**	**0**	**0.82**	**0.98**	**0**	**0**
					**0**	**0**	**0**	**0**	**4.35**	**0**	**0**	**0**	**0**	**4.39**
					**98.89**	**98.21**	**87.95**	**95.21**	**92.21**	**86.74**	**98.04**	**95.59**	**76.72**	**95.82**

### Multivariate analysis of the volatile dataset from four *Jasminum* species

**Fig. 3:**
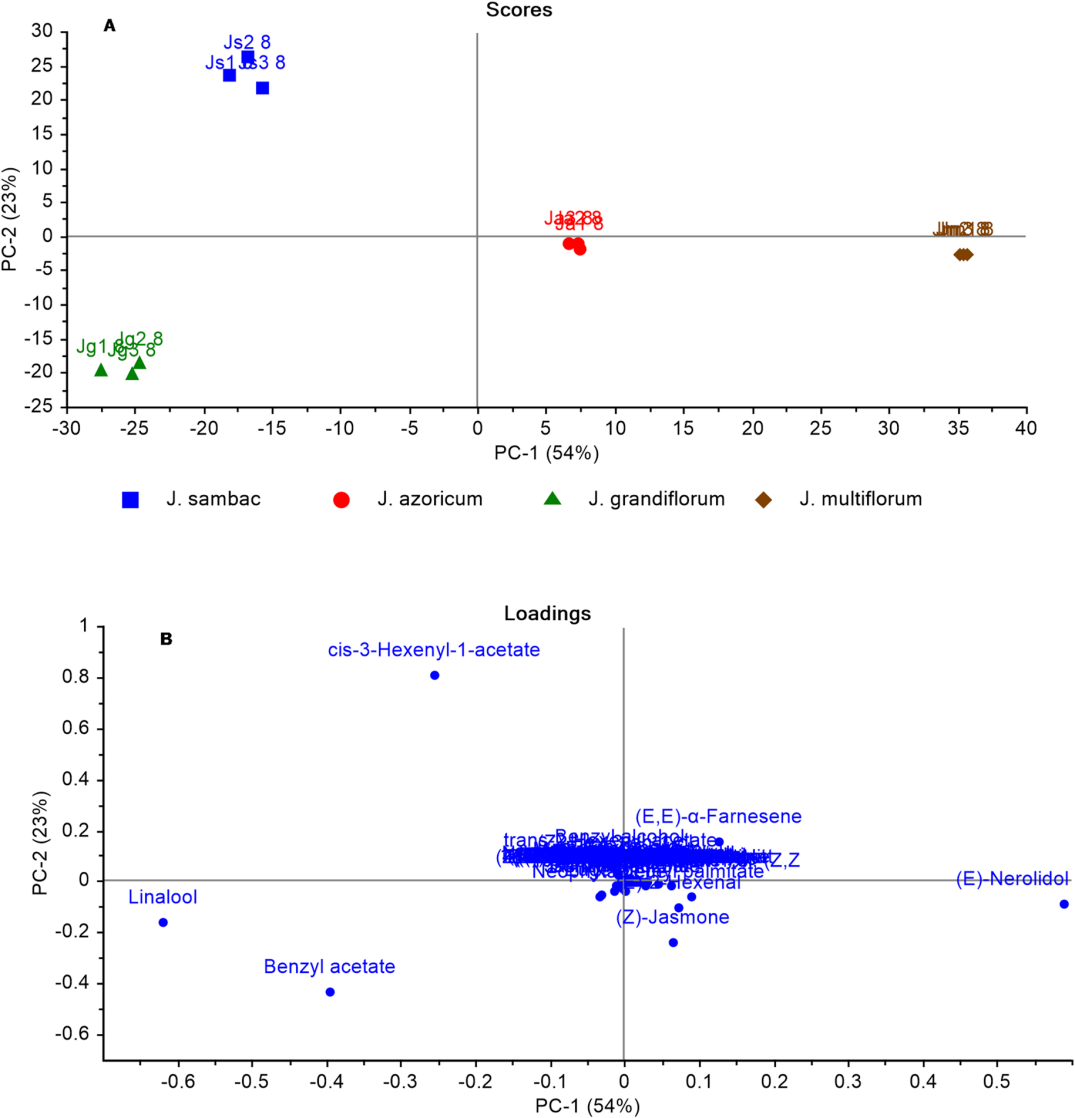
**(A)** Score plot of the volatile metabolites analyzed by Headspace-GC-MS of flowers of four Jasmine species collected in August using unsupervised principal component analysis (PCA). **(B)** Loading plot for PC1 and PC2 with associated contributing metabolites.

**Fig. 4:**
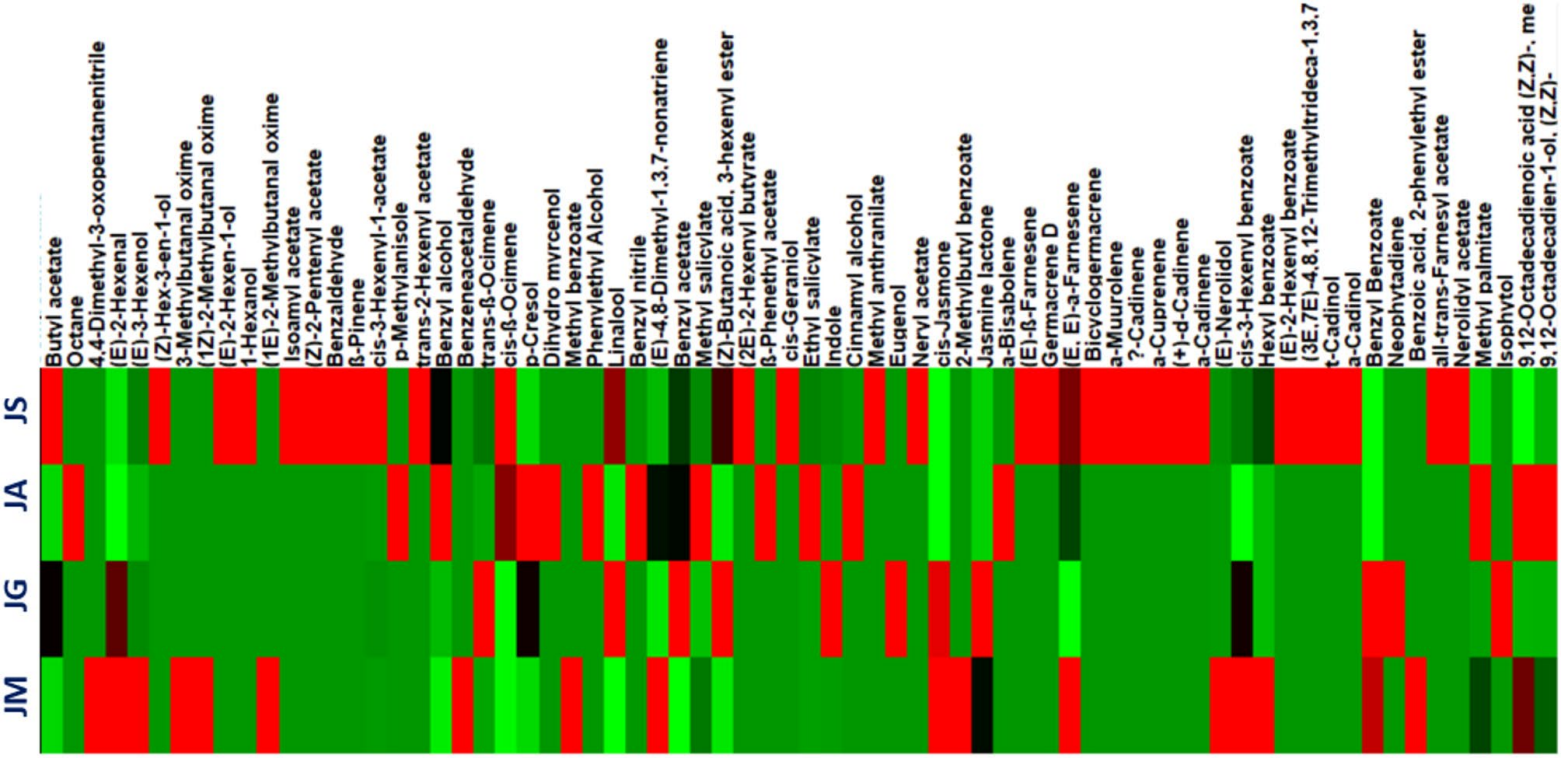
A heatmap comparison based on the abundance of individual components of headspace volatiles from JM, JG, JA, and JS collected in August; red indicates the highest levels of each identified component, and light green indicates the lowest levels.

The GC-MS-HS-based dataset was subjected to unsupervised multivariate data analysis techniques such as principal component analysis (PCA) and hierarchical clustering analysis (HCA). Multivariate data analysis was carried out in an untargeted way to accurately identify differences and similarities across specimens^89^. The first model (Fig. 3) integrated GC-MS data of headspace volatiles of flowers collected in August from four distinct jasmine species. A pair of orthogonal PCs was found to account for 77% of the variation when analyzed using the PCA model. PC1 accounted for 54% of the variance and PC2 for 23%, as illustrated in the PCA score plot (Fig. 3A). The PCA model displayed the segregation of *J. multiflorum* flowers collected in August (Jm 8-HS), which was found on the far-right side (positively to PC1 and negatively to PC2) and *J. azoricum* segregated directly to the center of the plot. *J. grandiflorum* flowers collected in August (Jg 8-HS) negatively contributed to PC1 and PC2. However, the remaining jasmine species (*J. sambac*), which had negative PC1 values, were arranged on the left side of the plot (Fig. 3A). The loading plot (Fig. 3B) explains the metabolites that contribute to this segregation. The elevated concentrations of benzyl acetate and linalool in Jg 8-HS were assumed to be the reason for their separation. Furthermore, there is a considerable accumulation of *cis*-3-hexenyl-1-acetate in Js 8-HS, which mediates its segregation. Ja 8-HS segregated negatively to PC2 and positively to PC1, where benzyl alcohol was abundant in this species and at a lower concentration in Js 8-HS. The reason for this separation of Ja 8-HS was attributed to its elevated amounts of benzyl alcohol and methyl palmitate. In contrast, the segregation of Jm 8-HS (positively to PC1 and negatively to PC2) can be attributed to *trans*-nerolidol, (*E*)-2-hexenal. *cis*-Jasmone was the major volatile fragrance characterizing *J. multiflorum* and *J. grandiflorum* and contributing to its separation on the lower side of the scoring plot, which was consistent with previous reports^16^. A heatmap displaying the abundance of individual components of headspace volatiles from *J. multiflorum*, *J. grandiflorum*,* J. azoricum*, and *J. sambac* is displayed in Fig. 4.

**Fig. 5:**
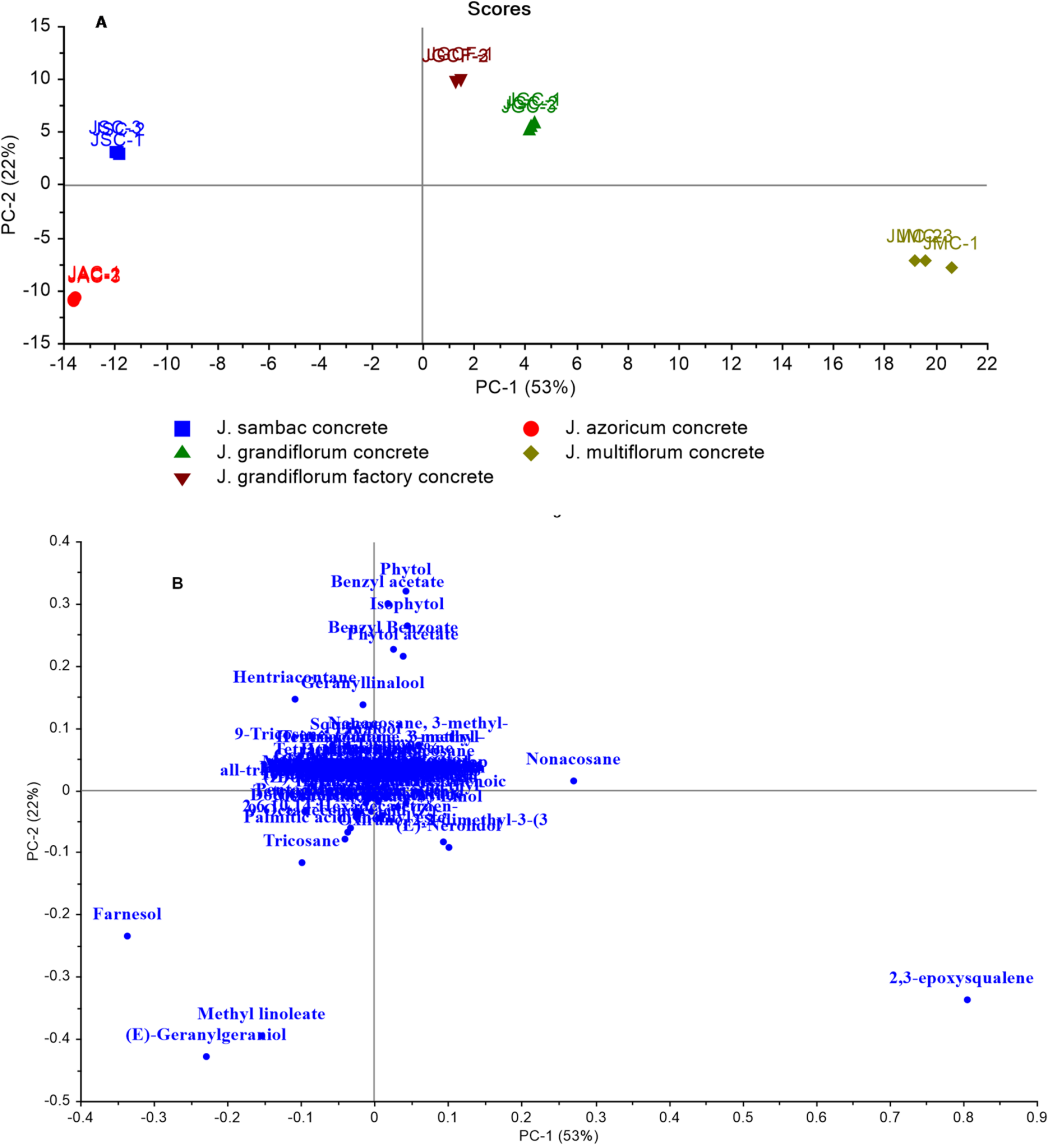
Principal component analysis of jasmine concrete volatiles composition as analyzed by GC-MS: **(A)** Score plot and **(B)** Loading plot for PC1 and PC2 with associated contributing metabolites.

Another model was developed using a GC-MS dataset of concrete volatiles from four *Jasminum* species, besides the purchased *J. grandiflorum* concrete (JGCF) (Fig. 5). In relation to PC1, the JSC and JAC were better separated and clustered together on the left, whereas the JGC, JMC, and JGCF were grouped on the right of PC1. JAC was segregated negatively to PC1 and PC2; this could be attributed to *trans-*geranylgeraniol and methyl linoleate. Contrariwise, (*Z*)-9-tricosene and hentriacontane, which were more abundant in JSC than other species, were responsible for JSC segregation (negative toward PC1 and positive toward PC2), as demonstrated by the loading plot (Fig. 5B). Additionally, farnesol was responsible for the separation of JSC and JAC from others, having a negative effect on PC1, and was exclusively abundant in JSC (9.96%) and JAC (9.5%). The segregation of the JMC in the far-right side of the score plot could be attributed to (*E*)-nerolidol and 2,3-epoxy squalene, as seen in the loading plot in Fig. 5B. The purchased *J. grandiflorum* concrete was close to the extracted *J. grandiflorum* concrete. PC2 separated JGC and JGCF, which were in the same quadrant; benzyl acetate was responsible for the segregation of the JGCF sample, positively contributing to PC1.

**Fig. 6:**
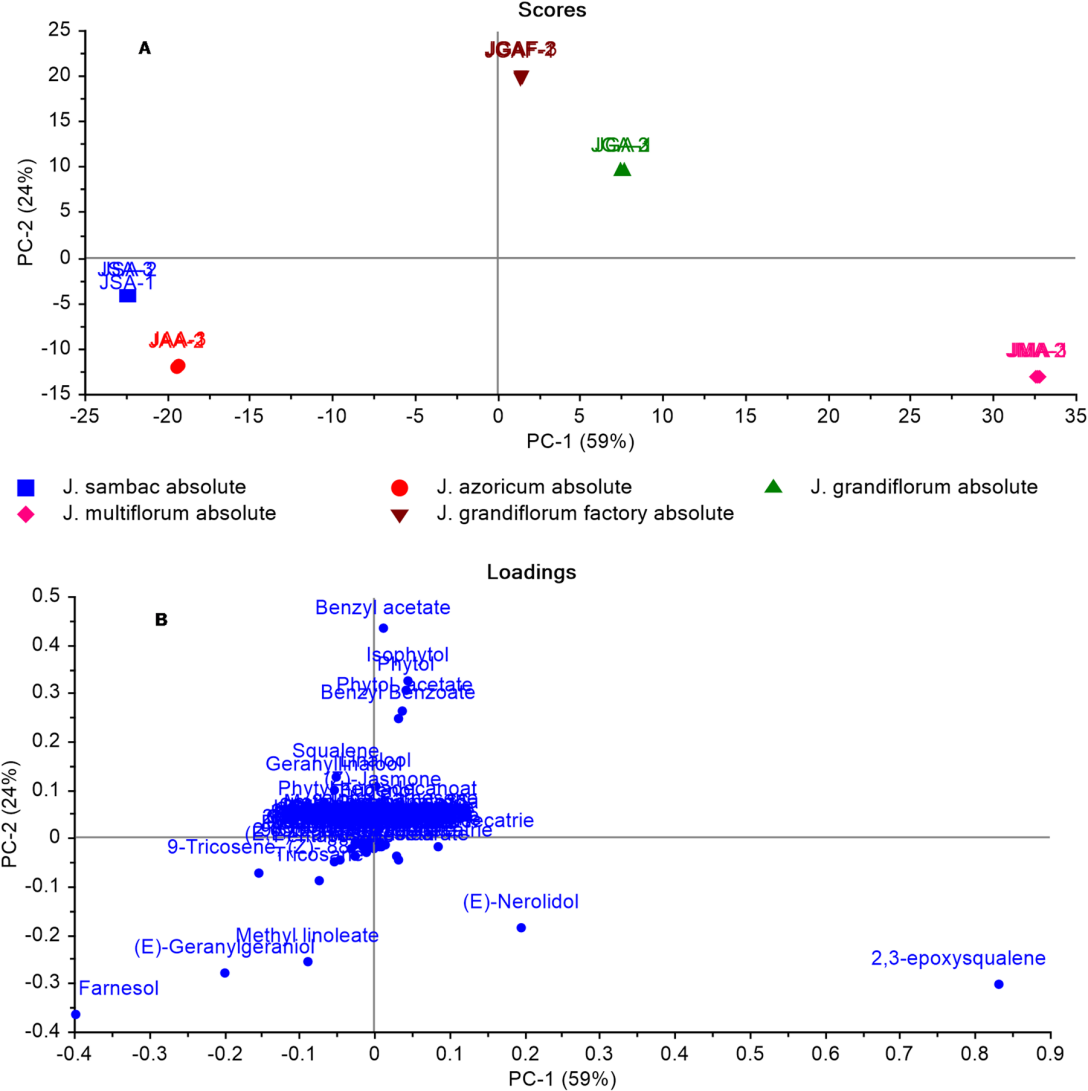
Principal component analysis of jasmine absolute volatiles composition as analyzed by GC-MS: **(A)** Score plot and **(B)** Loading plot for PC1 and PC2 with associated contributing metabolites.

GC-MS-based data of jasmine absolute samples were subjected to PCA (Fig. 6). The score plot (Fig. 6A) showed that JMA, JGA, and JGAF were positioned on the right side for absolute from flowers collected in August. In contrast, JSA and JAA were found on the left side. All JAA and JSA samples were grouped on the left side of the score plot with negative PC1 values, whereas JMA (positively to PC1 and negatively to PC2) and JGA and JGAF were clustered together in the top right quadrant (positively to PC1 and PC2). The loading plot (Fig. 6B) showed that (*Z*, *E*)-farnesol was the primary metabolite responsible for the segregation of JSA and JAA within the same quadrant (negatively to PC1 and PC2). Additionally, JAA was differentiated from JSA owing to the greater abundance of *trans*-geranylgeraniol and methyl linoleate in JAA. The loading plot in Fig. 6B illustrates how *trans*-nerolidol and 2,3-epoxy squalene were responsible for the segregation of JMA on the far-right side of the score plot. Benzyl acetate, benzyl benzoate, isophytol, phytol, and phytol acetate were responsible for the segregation of the JGA and JGAF in the upper-right section of the score plot (positively to PC1 and PC2). JGA and JGAF were in the same quadrant, where PC2 separated them, with benzyl acetate being a significant volatile compound in the JGAF sample.

**Fig. 7:**
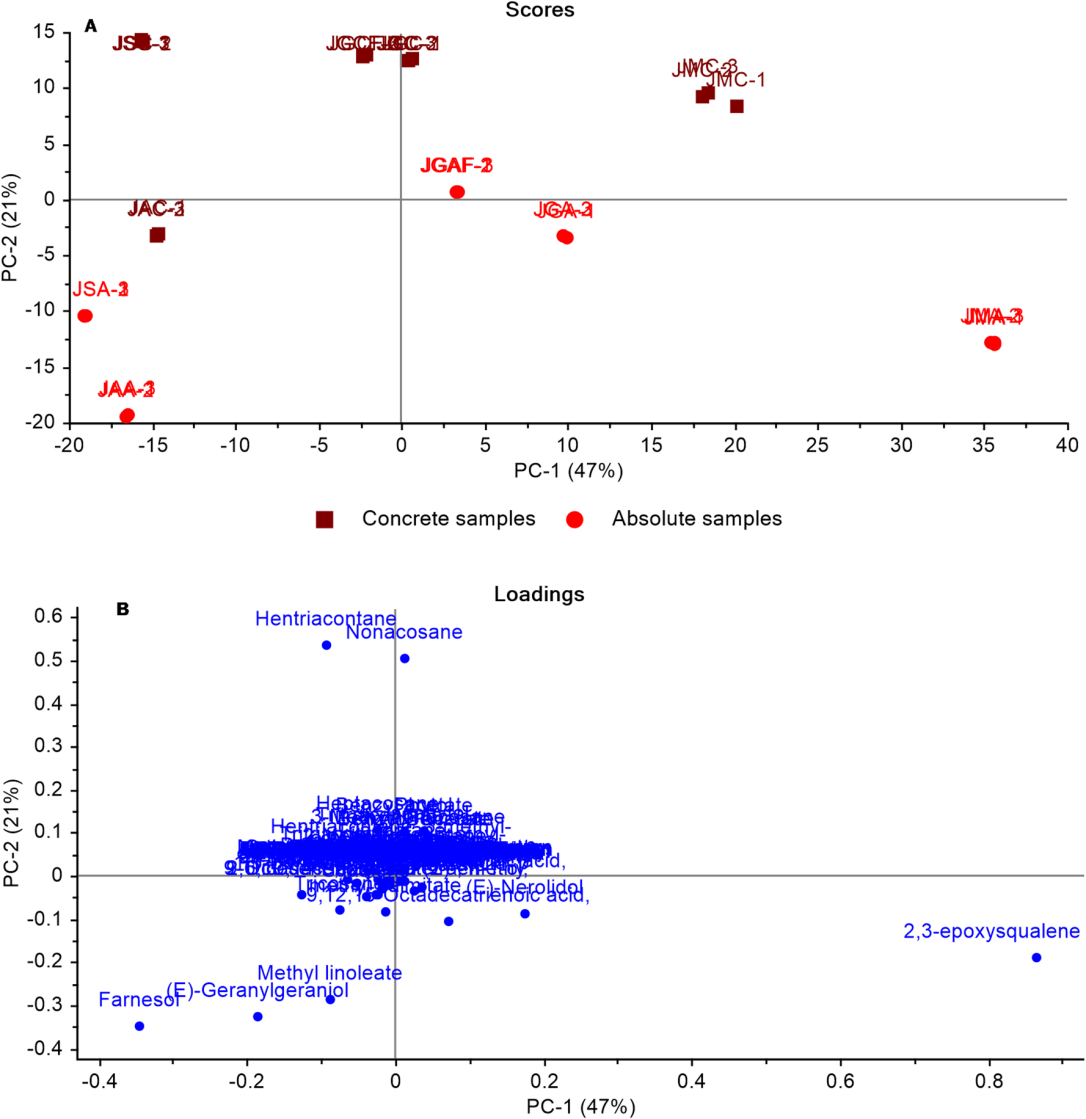
Principal component analysis of jasmine concrete along with absolute volatiles composition as analyzed by GC-MS: **(A)** A score plot and **(B)** Loading plot for PC1 and PC2 with associated contributing metabolites.

The constructed model (Fig. 7) combined GC-MS data from concrete and absolute volatiles. The model provided two orthogonal PCs that explained 68% of the total variation in the data (PC1 accounted for 47% and PC2 for 21%), as shown in (Fig. 7A). All of the concrete samples were segregated from the absolute volatiles along PC2, with the concrete samples clustered on the upper side of the score plot positively to PC2 and the absolute volatiles clustered on the lower side of the score plot negatively to PC2. The high levels of hentriacontane and nonacosane in concrete were shown to cause separation as illustrated in the loading plot (Fig. 7B). (*E*)-Geranylgeraniol, methyl linoleate, and farnesol were the major metabolites responsible for the segregation of the JAA and JSA from JAC and JSC, which were more abundant in absolute volatiles.

Another model (Supp. Fig. S1) incorporated GC-MS data from concrete and absolute volatiles alongside headspace volatiles. The PCA model identified two orthogonal PCs that explained 49% of the variation. Figure S1A displays the PC1/PC2 score plot, with concrete and absolute oil samples on the right side with positive PC1 values and the headspace flower volatiles on the left with negative PC1 values. The loading plot (Supp. Fig. S1B) can help clarify the segregation profile in the PCA score plot by identifying the attributed metabolites. The segregation of Jg 8-HS, Js 8-HS, Ja 8-HS, and Jm 8-HS flowers (negatively to PC1) was explained by the higher abundance of linalool, *cis*-3-hexenyl-1-acetate, benzyl acetate, and *cis*-jasmone in flowers compared to absolute and concrete volatiles (Supp. Fig. S1B). Additionally, 2,3-epoxysqualene, nonacosane, hentriacontane, farnesol, and (*E*)-geranylgeraniol were responsible for the segregation of jasmine concrete and absolute on the right side of the score plot (positively to PC1).

HCA was used to analyze the headspace volatiles, concrete, and absolute of the four *Jasminum* species collected in August, yielding two significant clusters as seen in (Supp. Fig. S1C). Cluster (1a) includes *J. grandiflorum* and *J. sambac* flowers, while cluster (1b) includes all jasmine (concrete, absolute, factory products, *J. azoricum* and *J. multiflorum*). The presence of *cis*-3-hexenyl-1-acetate (Table S1) differentiated *J. sambac* from *J. grandiflorum* within cluster (1a), explaining its grouping away from the other jasmine species (Supp. Fig. S1C).

### The impact of the month of collection on volatile datasets of four *Jasminum* species

Principal Component Analysis of headspace volatiles of *Jasminum* flowers Js 7-HS and Js 8-HS collected in July and August provided a PCA model comprising two orthogonal PCs (Supp. Fig. S2) with total variance coverage of 99% (Supp. Fig. S2A). The score plot (Supp. Fig. S2A) revealed Js 7-HS being positioned on the right side, whereas on the left side, Js 8-HS were located. The loading plot (Supp. Fig. S2B) showed that Js 8-HS had higher levels of benzyl acetate and *cis*-3-hexenyl-1-acetate, indicating a preference for a sweet fruity aroma compared to other time periods. The *cis*-3-hexenyl-1-acetate content was around 33%, whereas it was missing in Js 7-HS. In contrast, *cis*-*β*-ocimene and linalool were the main metabolites responsible for the segregation of the Js 7-HS, positively contributing to PC1(Supp. Fig. S2B). Linalool, a popular flavor and fragrance molecule, is a monoterpene alcohol found naturally in fragrant plants^90^. The main component of Js 7-HS and Js 8-HS, linalool, was found to be 63.06% and 25.41%, respectively.

A PCA model with two orthogonal PCs (Supp. Fig. S3) and a 97% variance coverage was constructed by principal component analysis of Ja 6-HS, Ja 7-HS, and Ja 8-HS prepared throughout a range of months (Supp. Fig. S3A). Fig. S3A showed the PC1/PC2 score plot in which the oil samples of Ja 6-HS and Ja 7-HS were located on the right side with positive PC1 values, whereas the Ja 8-HS were positioned on the left side of the plot with negative PC1 values. Benzyl acetate was responsible for the segregation of Ja 6-HS (negatively to PC2) and Ja 8-HS (negatively to PC1) at 8.16% and 10.57%, respectively. Furthermore, Ja 6-HS and Ja 8-HS contained larger concentrations of benzyl acetate than Ja 7-HS. (*E*,* E*)-*α*-Farnesene and *α*-bisabolene were responsible for the segregation of Ja 8-HS in the upper left side of the plot. The loading plot (Supp. Fig. S3B) revealed that phenylethyl alcohol and benzyl alcohol were the major metabolites responsible for Ja 7-HS segregation (positively for PC1 and PC2). Furthermore, there is a significant accumulation of methyl linolenate in Ja 6-HS, which promoted its segregation, accounting for 7.68%.

GC-MS-HS-based data of Jg-HS were subjected to PCA (Supp. Fig. S4), and the score plot (Supp. Fig. S4A) revealed that Jg 7-HS and Jg 8-HS were positioned on the right side. In contrast, Jg 6-HS was found on the left side (negatively to PC1 and PC2). The PCA model revealed two orthogonal PCs that accounted for 98% of the variance. The loading plot (Supp. Fig. S4B) revealed that linalool was the major metabolite responsible for Jg 8-HS segregation (positively to PC1 and PC2), accounting for 14.03%, 21.71%, and 38.19% of Jg 6-HS, Jg 7-HS, and Jg 8-HS, respectively. In contrast to *J. grandiflorum* collected in July and August, which had an abundance of 0.82% and 0.98% of indole, respectively, *J. sambac* had a lower abundance of 0.3%.

Based on headspace analysis, the most prevalent class among the four jasmine species was alcohol. Results observed herein were in agreement with previous findings, where *β*-linalool was the most common alcohol, constituting 58, 7, 8, and 11% in Egyptian *J. grandiflorum* flower HS, *J. sambac* flower HS, and *J. grandiflorum* absolutes and concretes^16^. The segregation of Jg 7-HS on the right side of the scoring plot (positively to PC1 and negatively to PC2) can be related to benzyl acetate, which was more abundant in July than in August or June, accounting for 47.68%, 23.32%, and 4.29%, respectively. The abundance of benzyl acetate in jasmine flowers was in agreement with previous reports, contributing to its very sweet and calming scent^65^. Furthermore, Jg 6-HS was distinguished from Jg 7-HS and Jg 8-HS by the higher abundance of (*E*)-2-hexenal and isophytol in Jg 6-HS. (*E*)-2-Hexenal accounted for 27.5% of Jg 6-HS, while isophytol accounted for 7.99%.

A separate model was developed utilizing the GC-MS-HS-based dataset, including seasonal variation of Jm 7-HS and Jm 8-HS (Supp. Fig. S5). The PCA model identified two orthogonal PCs that explained 98% of the variation. The score plot (Supp. Fig. S5A) indicated that Jm 7-HS was on the right side (positively to PC1 and negatively to PC2). In contrast, Jm 8-HS was detected on the left side (negatively to PC1 and PC2). Jm 7-HS segregation could be attributed to (*E*)-nerolidol and (*Z*)-jasmone, which were more abundant in Jm 7-HS than Jm 8-HS, as illustrated by the loading plot (Supp. Fig. S5B). In Jm 7-HS, (*E*)-nerolidol and (*Z*)-jasmone accounted for 45.49% and 15.18%, respectively, whereas in Jm 8-HS, they were 37.81% and 11.62%, respectively.

Js 8-HS and Ja 8-HS, as well as Jg 7-HS and Jg 8-HS, showed greater amounts of benzyl acetate than other periods of time, indicating a preference for sweet, fruity fragrances. Furthermore, linalool and eugenol levels were higher in Jg 8-HS than in other months. *α*-Farnesene was also more abundant in Js 8-HS. These findings are consistent with research showing that jasmine flowers cultivated in Egypt and China that were harvested in August produce a higher quality of essential oil than those harvested in autumn and spring, ^16,64^.

The volatile compounds in the four jasmine species varied significantly across months, as illustrated in (Table 2). Because of oxidation, photolysis, and other processes, several identified volatile chemicals, such as monoterpenoids, have been reported to be unstable^91^. It’s also important to note that the conditions utilized for even maceration, particularly during solvent evaporation, may cause chemical alterations to volatile compounds^61^. This might explain why flowers, concrete and absolute, have different levels of benzyl acetate, linalool and other volatiles. High temperatures produce extracts with less volatile components, but thermal degradation can damage aromatics^92^. HS is generally a quick, affordable, solvent-free method that may be used to analyze volatile substances^61^. Finally, Jasmine components were greatly impacted by the time of collection, the flower harvesting stage, and the extraction procedure, in agreement with previous reports^16,61,67^.

### In vitro monoamine oxidase (MAO-A) Inhibition assay

#### Inhibition of monoamine oxidase A (MAO-A) by Jasmine concrete

The volatile components of jasmine concrete and absolute have become more valuable in both medicine and industry due to their multipurpose qualities, which have sparked interest in their industrial and biological applications. According to our knowledge, this is the first report on the anti-MAO-A ability of hexane extracts (concrete) derived from four distinct jasmine species. Results showed that the tested concrete samples exhibited significant MAO-A inhibitory activity. The IC_50_ values varied from 4.008 to 35.39 µg/mL, with *J. grandiflorum* concrete showing the highest inhibitory activity. *J. grandiflorum* concrete displayed a significant inhibitory activity (IC_50_ = 4.008 µg/mL) compared to the purchased *J. grandiflorum* factory concrete, which had an IC_50_ of 21.41 µg/mL. *J. grandiflorum* concrete exhibited considerable activity, compared to the standard drug clorgyline (IC_50_ = 0.172 µg/mL). The second most potent jasmine concrete was *J. multiflorum*, with an IC_50_ of 7.681 µg/mL. *J. azoricum* concrete inhibited MAO-A enzyme with an IC_50_ value of 35.39 µg/mL, while *J. sambac* concrete had a higher IC_50_ value of 14.02 µg/mL, as shown in (Supp. Fig. S6), Table 3. Interestingly, the hydro-methanolic (HME) and boiling water (BWE) extracts of dried *J. grandiflorum* flower buds were previously tested for their efficiency against CNS diseases in vitro by assessing the inhibitory activity of monoamine oxidase A (MAO-A). It was noticed that both extracts demonstrated MAO-A inhibitory action, with IC_50_ levels of 603.16 µg/mL (HME) along with 699.74 µg/mL (BWE), whereas the reference drug (clorgyline) displayed an IC_50_ value > 0.012 µg/mL. This was the only report that could be traced regarding *J. grandiflorum’s* potential to inhibit MAO-A ^55^.

**Table 3:** The IC_50_ values for the enzyme inhibitory activities of Jasmine concrete, absolute and Clorgyline on monoamine oxidase (MAO)-A.

Jasminum species	IC_50_ values (µg/mL)
JSC	14.02 ± 0.473
JAC	35.39 ± 1.194
JGC	4.008 ± 0.135
JMC	7.681 ± 0.259
JGCF	21.41 ± 0.723
JSA	6.809 ± 0.295
JAA	15.82 ± 0.686
JGA	1.368 ± 0.059
JMA	1.047 ± 0.045
JGAF	0.463 ± 0.02
Clorgyline	0.172 ± 0.004

#### Inhibition of monoamine oxidase A (MAO-A) by Jasmine absolute

All tested *Jasminum* species absolute exhibited higher MAO-A inhibitory activity compared to the concrete samples (Table 3). Purchased *J. grandiflorum* absolute showed promising anti-MAO-A activity, with an IC_50_ of 0.463 µg/mL, demonstrating considerable potency in comparison to the standard anti-MAO-A medication clorgyline, which exhibited an IC_50_ of 0.172 µg/mL. Furthermore, promising anti-MAO-A activity was shown by *J. grandiflorum* absolute and *J. multiflorum* absolute, with IC_50_ values of 1.368 and 1.047 µg/mL, respectively, demonstrating notable potency in comparison to clorgyline. MAO-A was likewise suppressed by *J. sambac* and *J. azoricum* absolute, with IC_50_ values of 6.809 and 15.82 µg/mL, respectively, as shown in (Supp. Fig. S7), Table 3.

The MAO-A inhibitory activity of jasmine concrete, and absolute could be attributed to their components. Eugenol and (*E*, *E*)-*α*-farnesene were reported to inhibit monoamine oxidase A and exhibit antidepressant-like activity^34,53^. Besides, the identified volatile components of concrete and absolute exhibited noteworthy in vitro MAO-A inhibitory activity.

Previous studies reported that methyl jasmonate exhibited antidepressant-like effects in LPS-treated mice, suggesting a potential role in neuropsychiatric research^40^. The study conducted by Umukoro et al.. reported that methyl jasmonate reduced immobility in mice during forced swim (FST) and tail suspension tests (TST), indicating a possible antidepressant-like effect in preclinical models^50^.

Several studies have shown that inhaling the scent of jasmine tea with *J. sambac* blossoms has sedative impacts on autonomic nerve activity and mood. Linalool, one of the primary odor components of jasmine tea, is responsible for these effects^93^. Sesquiterpene alcohol constituents identified in jasmine concrete and absolute, such as *trans*-nerolidol (3.99 and 11.94%), have been proven to have biological activity, exhibiting antidepressant effect^94^. Furthermore, nerolidol showed strong anti-neuroinflammatory and antioxidant activity. Nerolidol enhanced locomotor activity, reduced motor incoordination and memory impairment, and decreased oxidative/nitrosative stress, exhibiting promising neuroprotective benefits^95^. In addition, benzyl acetate, linalool, farnesol, and nerolidol have been found to protect neuronal cells from oxidative stress, neuroinflammation, and death^30,96–98^. Phytol demonstrated a strong antidepressant-like effect by shortening the duration of immobility in the forced swimming test, decreasing lipid peroxidation and nitrite levels, and raising catalase and superoxidase activity in specific mouse brain regions^33^. Furthermore, phytol was reported to exhibit anti-insomnia and calming properties, and might boost GABA levels by inhibiting succinic semialdehyde dehydrogenase (SSADH), a GABA degradative enzyme^99^. Moreover, plant-derived diterpenes have demonstrated antidepressant effects and antioxidant capacity. Phytol, a diterpene alcohol identified in *J. grandiflorum*, was reported to reduce immobility in the forced swim test, suggesting a potential antidepressant-like effect in preclinical models^33^.

### Correlation of chemical composition with MAO-A inhibitory actions

**Fig. 8:**
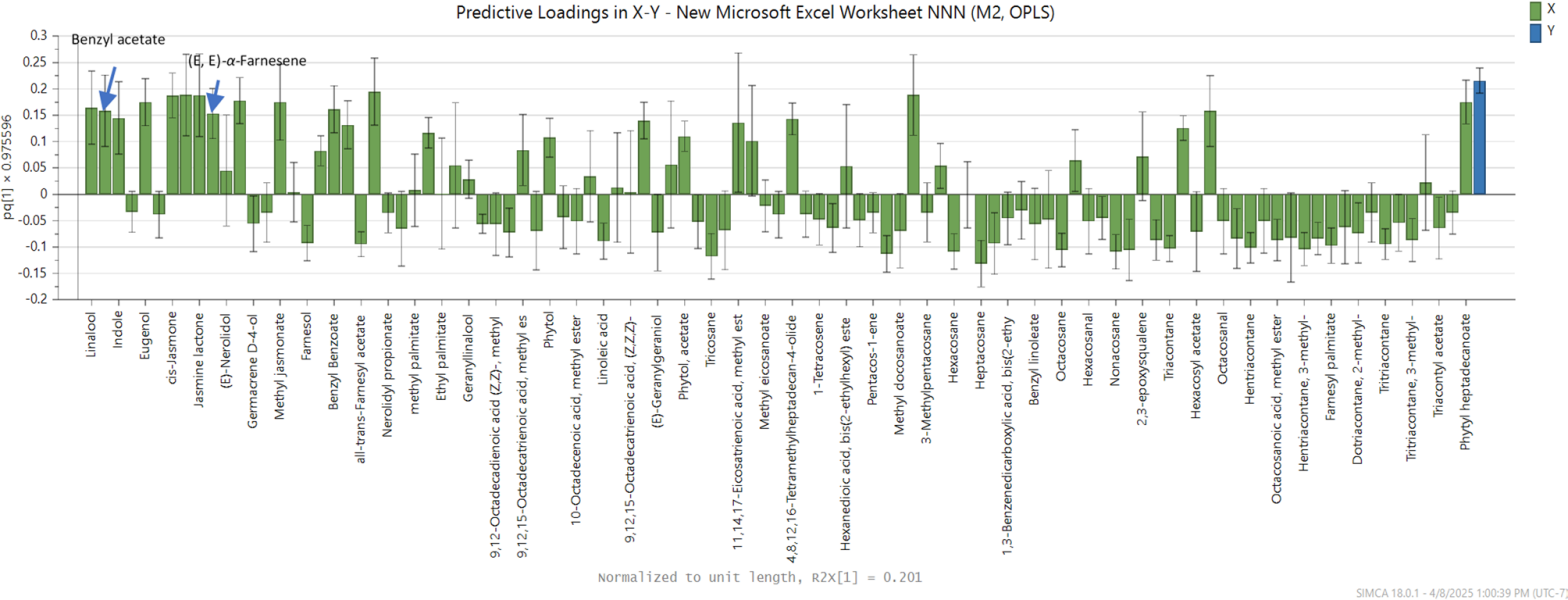
OPLS-derived loading column graph of volatile abundance versus MAO-A inhibitory 1/IC_50_ with attributed compounds.

Orthogonal Partial Least Squares (OPLS) regression analysis was employed to link chemical variability with biological activity and to identify the volatile compounds responsible for the observed effects. The X variable represented the abundance of volatile constituents, while the Y variable corresponded to the MAO-A inhibitory activity expressed as 1/IC_50_ values. The OPLS score plot explained 98.9% of the total variation in Y (R²Y = 0.989) and exhibited very good predictive ability 98.4% (Q² = 0.984), which means the model is very strong and reliable. Permutation testing (100 permutations) confirmed robustness, with significantly lower R^2^ (0.249) and Q^2^ (-0.667) values compared to the original model. In addition, the OPLS model’s Cross Validation-Analysis of Variance (CV-ANOVA) resulted in statistical significance (*p* = 1.47 × 10⁻^19^), indicating its robustness and dependability. The loading column plot (Fig. 8) revealed that linalool, indole, benzyl acetate, eugenol, (*E*, *E*)-*α*-farnesene, methyl jasmonate, and phytol were positively correlated with the biological activity. Additionally, the correlation between X and Y variables was evaluated using the Variable Importance in the Projection (VIP) plot (Supp. Fig. S8), which confirmed these constituents as major contributors to the biological activity with respective VIP scores of 1.71, 1.50, 1.64, 1.81, 1.59, 1.81, and 1.11, respectively. However, these findings do not exclude the possibility of synergistic interactions among volatile oil constituents influencing the MAO-A inhibition of *Jasminum* samples. In fact, therapeutic effects in natural products are often the result of complex synergy among multiple metabolites, rather than the action of a single compound^100^.

## Materials and methods

### Plant material

Fresh *J. grandiflorum* flowers were collected from a farm in Shubra Belola, Qotour, Gharbia, Egypt (coordinates: 30.956243, 30.938555) in the early mornings of June, July, and August of 2022. Additionally, fresh blossoms of *J. azoricum* were picked early in the mornings of June, July, and August from Banha-Al Qalyubia, Egypt (coordinates: 30.497622, 31.194529). Besides, flowers from *J. multiflorum* and *J. sambac* were collected at the early mornings in July and August of 2022 from fields in Bagour, Menofia, Egypt (coordinates: 30.4184549, 31.0356259), and the medicinal plants research station in the Faculty of Pharmacy, Ain Shams University, Cairo, Egypt (coordinates: 30.074079, 31.292067), respectively. The collection adhered to the IUCN Policy Statement on Research Involving Species at Risk of Extinction, and all collection requirements were followed to ensure compliance with institutional, national, and international guidelines and legislation. All jasmine blooms were authenticated by Mrs. Therese Labib, a consultant for the Ministry of Agriculture and the former head of Orman Botanical Garden. *J. grandiflorum* concrete and absolute were obtained from Egypt Aromatic for Essential Oil & Aromatic Products factory in Shubra Belola, Qotour, Gharbia, Egypt. Plant material and products received a code as listed in Table 4. Voucher specimens were deposited at the herbarium of the Pharmacognosy Department, Faculty of Pharmacy, Ain Shams University in Cairo, for plant identification and categorization (PHG-P-JS-494, PHG-P-JA-496, PHG-P-JG-340, PHG-P-JM-495).

### Preparation of Jasmine concrete and absolute

Fresh flowers of the Jasmine species harvested early in the morning in August were extracted three times separately using hexane to create jasmine concrete based on the previously reported method^16^. Briefly, 10 g of freshly opened flowers were soaked in 100 mL of hexane and shaken for 1 h on a SCILOGEX SK-O330-Pro Digital Orbital Shaker, 100–500 rpm, USA. The extracts were filtered, and the solvent was subsequently evaporated under vacuum using a BUCHI R-300 EL Rotavapor w/ I-300 Pro, F-305 Chiller & Condenser, Switzerland, at a maximum temperature of 40 °C to produce jasmine concrete. The absolute was retrieved from concrete following the method described by Tamogami et al. ^83^. Jasmine absolute is extracted from concrete using 95% ethanol, washed three times, and filtered at -80 °C to remove wax. Jasmine absolute is produced by evaporating alcohol at temperatures below 40 °C.

**Table 4:** Jasmine samples, codes, and the source of collection during various harvesting months.

NO	Sample Codes	Sample Name	Source (Geographical Origin)	Month of Collection
1	JSC	Jasminum sambac Concrete	ASU medicinal plant station-Cairo	Aug-22
2	JAC	*Jasminum azoricum* Concrete	Banha-Al Qalyubia	Aug-22
3	JGC	*Jasminum grandiflorum* Concrete	Shubra Belola-Gharbia	Aug-22
4	JMC	*Jasminum multiflorum* Concrete	Bagour-Menofia	Aug-22
5	JGCF	*Jasminum grandiflorum* Concrete-Factory	Shubra Belola-Gharbia	Aug-22
6	JSA	*Jasminum sambac* Absolute	ASU medicinal plant station -Cairo	Aug-22
7	JAA	*Jasminum azoricum* Absolute	Banha-Al Qalyubia	Aug-22
8	JGA	*Jasminum grandiflorum* Absolute	Shubra Belola-Gharbia	Aug-22
9	JMA	*Jasminum multiflorum* Absolute	Bagour-Menofia	Aug-22
10	JGAF	*Jasminum grandiflorum* Absolute-Factory	Shubra Belola-Gharbia	Aug-22
11	Js 7-HS	*Jasminum sambac* flower-July Headspace	ASU medicinal plant station -Cairo	Jul-22
12	Js 8-HS	*Jasminum sambac* flower-August-Headspace	ASU medicinal plant station -Cairo	Aug-22
13	Ja 6-HS	*Jasminum azoricum* flower-June-Headspace	Banha-Al Qalyubia	Jun-22
14	Ja 7-HS	*Jasminum azoricum* flower-July-Headspace	Banha-Al Qalyubia	Jul-22
15	Ja 8-HS	*Jasminum azoricum* flower-August- Headspace	Banha-Al Qalyubia	Aug-22
16	Jg 6-HS	*Jasminum grandiflorum* flower-June Headspace	Shubra Belola-Gharbia	Jun-22
17	Jg 7-HS	*Jasminum grandiflorum* flower-July-Headspace	Shubra Belola-Gharbia	Jul-22
18	Jg 8-HS	*Jasminum grandiflorum* flower-August-Headspace	Shubra Belola-Gharbia	Aug-22
19	Jm 7-HS	*Jasminum multiflorum* flower-July-Headspace	Bagour-Menofia	Jul-22
20	Jm 8-HS	*Jasminum multiflorum* flower-August-Headspace	Bagour-Menofia	Aug-22

ASU: Ain Shams University.

### GC-MS headspace analysis (HS)

A 5 mL glass vial of a Shimadzu headspace sampler HS-20 was filled with 3 g of fresh jasmine flower. A gas chromatograph mass spectrometer (GC-MS-QP2020) from Shimadzu (Kyoto, Japan) with a splitless mode and RTX™-1 MS column (30 m × 0.25 mm id. × 0.25 μm film thickness) from Restek (Bellefonte, PA, USA) was interfaced with a Shimadzu HS-20 headspace sampler. The temperature of the sample line and transfer line was maintained at 150 °C, while the oven was set at 80 °C. The following settings were used for the headspace sampling: 8 min for equilibration, 2 min for pressurization, and 5 min for needle flushing. The splitless injection technique was employed to analyze materials that had lower volatile contents. The following conditions were applied for GC-MS headspace analysis: the temperature of the column oven was kept at 45 °C for two minutes, then raised to 300 °C (5 °C/min) and maintained there for 5 min; the carrier gas was helium, flowing at a rate of 1.41 mL/min; APCI pressure: 50 kPa; the ion source temperature is 200 °C, whereas the interface temperature is 280 °C.

### GC-MS analysis of Jasmine concrete and absolute

The jasmine concrete and absolute were analyzed using a Shimadzu QP2010 gas chromatograph with a quadrupole mass spectrometer (Kyoto, Japan). The gas chromatograph employed an Rtx-5MS fused bonded column (30 m × 0.25 mm id. × 0.25 μm film thickness) from Restek (Bellefonte, PA, USA) with a split-splitless injector. A volume of 1 µL of the diluted sample (1% *v/v*) in hexane was injected in split mode with a split ratio of 1:30, and helium was used as the carrier gas at a flow rate of 1.37 mL/min. The injector temperature was set to 280 °C, while the oven temperature was maintained at 50 °C for 3 min. After that, the temperature gradually increased to 300 °C at a rate of 5 °C/min and was maintained at this temperature for 10 min. MS operational conditions include an ion source temperature of 220 °C, electron ionization (EI) mode at 70 eV, using a filament emission current of 60 mA, and scanning from 35 to 500 amu. AMDIS software (www.amdis.net) was employed for the deconvolution of peaks. Volatile components were identified by comparing their retention indices (RI) and mass spectra (MS) with those listed in the NIST mass spectral library database, Adams^101^, and the literature^102–105^. The retention indices were calculated relative to a series of standard *n*-alkanes (C8-C28) injected under the same conditions. Results were processed using GCMSsolution Workstation Software for Gas Chromatography-Mass Spectrometry.

### Multivariate data analysis

Unsupervised multivariate data analysis was performed using the Unscrambler X10.3 CAMO software (Computer Aided Modelling, AS, Norway). Principal component analysis (PCA) and hierarchical cluster analysis (HCA) were used to provide an overview of sample variation and volatile composition differences, while HCA was used to identify similarities and differences among the oils. The cluster analysis was conducted using Ward’s approach, and distances between clusters were computed using the squared Euclidean method^106–108^. Orthogonal Partial Least Squares (OPLS) analysis was conducted using SIMCA software (version 16.0.1, Umetrics, Sartorius-Stedim Data Analytics, Umeå, Sweden) to pinpoint the metabolites contributing to the observed biological activity^109^. Additionally, a heat map was generated using GC data processed with Hierarchical Clustering Explorer 3.5 software (Human-Computer Interaction Laboratory, University of Maryland, College Park, MD, USA)^110^.

### Monoamine oxidase (MAO-A) Inhibition assay

The inhibitory activity of *Jasminum* concretes and absolutes against MAO-A was assessed using a fluorometric technique via a BioVision MAO-A Inhibitor Screening Kit (Cat. No. K796-100, BioVision, USA) and according to the manufacturer’s directions^111^.

For the assay, 10 µL of test samples (dissolved in 2% DMSO), clorgyline (positive control, 10 µM), and assay buffer (negative control) were first added to the wells of a black 96-well microplate. Then, 50 µL of recombinant MAO-A enzyme solution containing 49 µL of MAO-A assay buffer and 1 µL of diluted MAO-A Enzyme was dispensed into each well, and the plate was incubated for 10 min at 25 °C to allow inhibitor–enzyme interaction. The reaction was initiated by adding 40 µL of the MAO-A working solution (37 µL assay buffer, 1 µL substrate, 1 µL developer, and 1 µL OxiRed probe) to each well. The mixture was incubated for 30 min at the proper temperature. After that, the fluorescence was measured in a 96-well plate reader at λ _excitation (Ex) **/** emission (Em)_ = 535/587 nm at 25 °C for 10–30 min.

The slope values were taken for all samples (S) and including Enzyme Control (EC), by dividing the net relative fluorescent units ΔRFU values: (RFU_2_ – RFU_1_) by the time Δt (T_2_ – T_1_), and the relative inhibition% was calculated using the following equation:

% Relative inhibition = [(slope of EC - slope of S) / slope of EC] x 100%.

## Conclusions

Jasmine represents a significant export crop valued globally for its commercial products and flowers. This work offered a comparative and comprehensive profiling of the volatile profile of four Egyptian *Jasminum* species concrete and absolute, along with purchased *J. grandiflorum* (concrete and absolute). Besides, variations in the volatile profile of scents in four distinct types of jasmine flowers were investigated herein to verify that the exceptional aroma quality of *J. grandiflorum* is the reason it is utilized in perfumery all around the world. Unsupervised multivariate data analysis identified benzyl acetate, benzyl benzoate, phytol, linalool, isophytol, geranyl linalool, methyl linoleate, and eugenol as key phytomarkers responsible for the popular *Jasminum grandiflorum* fragrance. Moreover, results showed variations in the composition of aroma volatiles utilizing various extraction techniques. The HS technique was more effective than solvent extraction for extracting volatile chemicals. This is because the heat-free approach reduces the possibility of volatile component modifications and loss during solvent removal. Furthermore, variation based on the month of collection of the selected jasmine species was analyzed herein by HS. Results demonstrated that August represents the best time for collection. Besides, the anti-MAO activity of the volatile oil from distinct *Jasminum* species concrete and absolute was assessed. Results revealed a promising MAO-A inhibitory action in vitro, suggesting potential for future investigation in the management of depression. Linalool, indole, benzyl acetate, eugenol, (*E*, *E*)-*α*-farnesene, methyl jasmonate, and phytol were the primary contributors to the biological activity. However, additional in vivo studies and clinical investigations are required to confirm these preliminary findings.

## Supplementary Information

Below is the link to the electronic supplementary material.

Supplementary Material 1

## Data Availability

Data are available upon request from the first author.
