# Supplementary material for: Comparative analysis of flower volatiles from four Jasminum species growing in Egypt using multivariate analysis
Source: Sci Rep. 2026 Mar 11;16:8947. doi: 10.1038/s41598-026-39688-w (PMC12988214; doi:10.1038/s41598-026-39688-w)
Supplement: Supplementary file 1 — Supplementary Material 1 [file 41598_2026_39688_MOESM1_ESM.doc]

**Supplementary Material**

**Comparative analysis of flower volatiles from four *Jasminum* species growing in Egypt using multivariate analysis**

Mohamed S. Yassin^1^, Iriny M. Ayoub^1^, Sherweit H. El-Ahmady^1^ and Abdel Nasser B. Singab^1, 2,*^

^1^ Department of Pharmacognosy, Faculty of Pharmacy, Ain Shams University, Abbassia, Cairo 11566, Egypt

^2^ Center for Drug Discovery Research and Development, Faculty of Pharmacy, Ain Shams University, Abbassia, Cairo 11566, Egypt

**Corresponding author**

*Abdel Nasser B. Singab, PhD

Professor of Pharmacognosy, Faculty of Pharmacy, Ain Shams University, Abbassia, Cairo 11566, Egypt.

Email: [AbdelnasserSingab@pharma.asu.edu.eg](mailto:AbdelnasserSingab@pharma.asu.edu.eg)

**Table S1.** Phytochemical profiling of the headspace aroma of *Jasminum sambac* (JS), *Jasminum azoricum* (JA), *Jasminum grandiflorum* (JG), and *Jasminum multiflorum* (JM) flowers collected in August, as well as GC-MS analysis of concrete (C), absolute (A), and factory (F) products

| **Peak** | **RT** | **Compound Name** | **RI** | **RI(REF)** | **Head Space** | | | | **Concrete** | | | | | **Absolute** | | | | |
| --- | --- | --- | --- | --- | --- | --- | --- | --- | --- | --- | --- | --- | --- | --- | --- | --- | --- | --- |
|  |  |  |  |  | **JS** | **JA** | **JG** | **JM** | **JSC** | **JAC** | **JGC** | **JMC** | **JGCF** | **JSA** | **JAA** | **JGA** | **JMA** | **JGAF** |
|  | 4.329 | Butyl acetate | 806 | 804 | 0.2 | - | 0.07 | - | - | - | - | - | - | - | - | - | - | - |
|  | 4.341 | Octane | 807 | 801 | - | 4.35 | - | - | - | - | - | - | - | - | - | - | - | - |
|  | 4.424 | 4,4-Dimethyl-3-oxopentanenitrile | 810 | 817 | - | - | - | 0.87 | - | - | - | - | - | - | - | - | - | - |
|  | 4.909 | (*E*)-2-Hexenal | 827 | 827 | 0.35 | - | 4.28 | 7.9 | - | - | - | - | - | - | - | - | - | - |
|  | 5.178 | (*E*)-3-Hexenol | 837 | 836 | 0.14 | - | 0.12 | 1.7 | - | - | - | - | - | - | - | - | - | - |
|  | 5.247 | (*Z*)-Hex-3-en-1-ol | 840 | 840 | 2.15 | - | - | - | - | - | - | - | - | - | - | - | - | - |
|  | 5.381 | 3-Methylbutanal oxime | 845 | 858 | - | - | - | 0.38 | - | - | - | - | - | - | - | - | - | - |
|  | 5.446 | (1*Z*)-2-Methylbutanal oxime | 847 | - | - | - | - | 1.97 | - | - | - | - | - | - | - | - | - | - |
|  | 5.535 | (*E*)-2-Hexen-1-ol | 850 | 850 | 0.8 | - | - | - | - | - | - | - | - | - | - | - | - | - |
|  | 5.633 | 1-Hexanol | 854 | 854 | 0.36 | - | - | - | - | - | - | - | - | - | - | - | - | - |
|  | 5.646 | (1*E*)-2-Methylbutanal oxime | 854 | - | - | - | - | 1.17 | - | - | - | - | - | - | - | - | - | - |
|  | 5.808 | Isoamyl acetate | 860 | 860 | 0.03 | - | - | - | - | - | - | - | - | - | - | - | - | - |
|  | 6.963 | (*Z*)-2-Pentenyl acetate | 902 | 897 | 0.25 | - | - | - | - | - | - | - | - | - | - | - | - | - |
|  | 7.61 | Benzaldehyde | 926 | 926 | 0.09 | - | - | - | - | - | - | - | - | - | - | - | - | - |
|  | 9.316 | *β*-Pinene | 988 | 988 | 0.07 | - | - | - | - | - | - | - | - | - | - | - | - | - |
|  | 9.466 | *cis*-3-Hexenyl-1-acetate | 993 | 990 | 33.09 | - | 0.55 | - | - | - | - | - | - | - | - | - | - | - |
|  | 9.723 | p-Methylanisole | 1002 | 1003 | - | 0.67 | - | - | - | - | - | - | - | - | - | - | - | - |
|  | 9.729 | *trans*-2-Hexenyl acetate | 1003 | 997 | 2.39 | - | - | - | - | - | - | - | - | - | - | - | - | - |
|  | 9.956 | Benzyl alcohol | 1010 | 1008 | 4.76 | 13.43 | 1.04 | - | - | - | - | - | - | - | - | - | - | - |
|  | 9.974 | Benzeneacetaldehyde | 1010 | 1009 | - | - | - | 2.93 | - | - | - | - | - | - | - | - | - | - |
|  | 10.667 | *trans*-*β*-Ocimene | 1033 | 1032 | 0.02 | - | 0.26 | - | - | - | - | - | - | - | - | - | - | - |
|  | 11.004 | *cis*-*β*-Ocimene | 1044 | 1043 | 1.36 | 0.86 | - | - | - | - | - | - | - | - | - | - | - | - |
|  | 11.457 | *p*-Cresol | 1058 | 1057 | - | 2.55 | 0.94 | - | - | - | - | - | - | - | - | - | - | - |
|  | 11.608 | Dihydro myrcenol | 1063 | 1062 | - | 1.18 | - | - | - | - | - | - | - | - | - | - | - | - |
|  | 11.854 | Methyl benzoate | 1071 | 1070 | - | - | - | 0.19 | - | - | - | - | - | - | - | - | - | - |
|  | 12.33 | Phenylethyl Alcohol | 1086 | 1086 | - | 1.03 | - | - | - | - | - | - | - | - | - | - | - | - |
|  | 12.483 | Linalool | 1097 | 1097 | 25.41 | 2.19 | 38.19 | - | 0.73 | - | - | - | 1.76 | - | - | - | - | 4.35 |
|  | 12.529 | Benzyl nitrile | 1093 | 1094 | - | 3.55 | - | - | - | - | - | - | - | - | - | - | - | - |
|  | 13.114 | (E)-4,8-Dimethyl-1,3,7-nonatriene | 1111 | 1104 | 0.12 | 0.58 | - | 1.76 | - | - | - | - | - | - | - | - | - | - |
|  | 14.121 | Benzyl acetate | 1163 | 1162 | 8.95 | 10.57 | 23.32 | - | - | - | - | - | 9.39 | - | - | - | - | 17.93 |
|  | 14.908 | Methyl salicylate | 1169 | 1169 | 0.31 | 3.67 | - | 0.41 | - | - | - | - | - | - | - | - | - | - |
|  | 15.049 | (*Z*)-Butanoic acid, 3-hexenyl ester | 1174 | 1173 | 0.13 | - | 0.28 | - | - | - | - | - | - | - | - | - | - | - |
|  | 15.274 | (2*E*)-2-Hexenyl butyrate | 1181 | 1177 | 0.02 | - | - | - | - | - | - | - | - | - | - | - | - | - |
|  | 16.682 | *β*-Phenethyl acetate | 1228 | 1229 | - | 0.83 | - | - | - | - | - | - | - | - | - | - | - | - |
|  | 17.009 | *cis*-Geraniol | 1240 | 1239 | 0.11 | - | - | - | - | - | - | - | - | - | - | - | - | - |
|  | 17.232 | Ethyl salicylate | 1248 | 1249 | 0.1 | 2.07 | - | - | - | - | - | - | - | - | - | - | - | - |
|  | 18.141 | Indole | 1297 | 1295 | 0.03 | - | 0.98 | - | - | - | - | - | 0.47 | - | - | - | - | 0.71 |
|  | 17.98 | Cinnamyl alcohol | 1274 | 1270 | - | 1.1 | - | - | - | - | - | - | - | - | - | - | - | - |
|  | 19.493 | Methyl anthranilate | 1346 | 1346 | 1.12 | - | - | - | 0.42 | - | - | - | - | - | - | - | - | - |
|  | 19.871 | Eugenol | 1359 | 1359 | - | - | 2.63 | - | - | - | - | - | 0.26 | - | - | 1.05 | - | 1.33 |
|  | 19.953 | 2,7-Octadiene-1,6-diol, 2,6-dimethyl- | 1362 | 1367 | - | - | - | - | - | - | - | - | - | 2.09 | - | - | - | - |
|  | 20.599 | Neryl acetate | 1365 | 1365 | 0.18 | - | - | - | - | - | - | - | - | - | - | - | - | - |
|  | 21.009 | *cis*-Jasmone | 1400 | 1399 | - | - | 10.29 | 11.62 | - | - | 0.33 | - | 1.05 | - | - | 0.86 | 0.39 | 2.57 |
|  | 21.896 | 2-Methylbutyl benzoate | 1412 | 1409 | - | - | - | 0.76 | - | - | - | - | - | - | - | - | - | - |
|  | 22.711 | Jasmine lactone | 1444 | 1442 | - | - | 1.89 | 0.59 | - | - | - | - | - | - | - | - | - | 0.33 |
|  | 22.884 | *α*-Bisabolene | 1451 | 1443 | - | 5.29 | - | - | - | - | - | - | - | - | - | - | - | - |
|  | 22.907 | (*E*)-*β*-Farnesene | 1451 | 1457 | 0.31 | - | - | - | - | - | - | - | - | - | - | - | - | - |
|  | 23.42 | (*Z*, *E*)-α-Farnesene | 1492 | 1493 | - | - | - | - | - | - | - | - | - | - | - | - | - | 0.2 |
|  | 23.521 | Germacrene D | 1475 | 1475 | 0.32 | - | - | - | - | - | - | - | - | - | - | - | - | - |
|  | 23.765 | (*E*, *E*)-α-Farnesene | 1506 | 1506 | 10.45 | 7.53 | 2.73 | 13.54 | 1.24 | - | 0.72 | 1.16 | 1.13 | - | - | 0.8 | 1.8 | 1.99 |
|  | 23.919 | Bicyclogermacrene | 1491 | 1491 | 0.13 | - | - | - | - | - | - | - | - | - | - | - | - | - |
|  | 24.011 | *α*-Muurolene | 1495 | 1495 | 0.03 | - | - | - | - | - | - | - | - | - | - | - | - | - |
|  | 24.325 | *γ*-Cadinene | 1507 | 1507 | 0.36 | - | - | - | - | - | - | - | - | - | - | - | - | - |
|  | 24.502 | *α*-Cuprenene | 1514 | 1514 | 0.47 | - | - | - | - | - | - | - | - | - | - | - | - | - |
|  | 24.56 | (+)-*δ*-Cadinene | 1516 | 1516 | 0.16 | - | - | - | - | - | - | - | - | - | - | - | - | - |
|  | 24.927 | *α*-Cadinene | 1530 | 1534 | 0.03 | - | - | - | - | - | - | - | - | - | - | - | - | - |
|  | 25.165 | (*E*)-Nerolidol | 1562 | 1562 | 0.53 | - | - | 37.81 | 0.35 | - | - | 3.99 | - | - | - | - | 11.94 | 0.11 |
|  | 25.437 | *cis*-3-Hexenyl benzoate | 1573 | 1573 | 0.89 | 0.59 | 1.1 | 1.69 | 0.38 | - | - | - | 0.34 | - | - | - | 0.36 | 1.01 |
|  | 25.495 | Hexyl benzoate | 1553 | 1551 | 0.14 | - | - | 0.76 | - | - | - | - | - | - | - | - | - | - |
|  | 25.535 | Germacrene D-4-ol | 1577 | 1577 | - | - | - | - | 2.07 | - | - | - | - | 2.22 | - | - | - | - |
|  | 25.603 | (*E*)-2-Hexenyl benzoate | 1557 | 1556 | 0.19 | - | - | - | - | - | - | - | - | - | - | - | - | - |
|  | 25.938 | (3*E*,7*E*)-4,8,12-Trimethyltrideca-1,3,7,11-tetraene | 1570 | 1566 | 0.03 | - | - | - | - | - | - | - | - | - | - | - | - | - |
|  | 26.163 | Methyl N-acetylanthranilate | 1603 | 1607 | - | - | - | - | - | - | - | - | 0.18 | - | - | - | - | - |
|  | 27.22 | *τ*-Cadinol | 1622 | 1622 | 0.28 | - | - | - | - | - | - | - | - | - | - | - | - | - |
|  | 27.248 | Methyl jasmonate | 1649 | 1652 | - | - | - | - | - | - | - | - | 0.28 | - | - | - | - | 0.9 |
|  | 27.513 | *α*-Cadinol | 1635 | 1634 | 0.19 | - | - | - | - | - | - | - | - | - | - | - | - | - |
|  | 27.987 | (*Z*)-methyl epijasmonate | 1680 | 1684 | - | - | - | - | - | - | 0.52 | - | 0.13 | - | - | 0.9 | - | - |
|  | 28.901 | Farnesol | 1718 | 1721 | - | - | - | - | 9.96 | 9.5 | - | - | - | 21.26 | 17.04 | - | 0.19 | - |
|  | 29.166 | (3*E*,10*Z*)-Oxacyclotrideca-3,10-diene-2,7-dione | 1729 | 1737 | - | - | - | - | - | - | - | - | - | - | - | 0.34 | - | 0.09 |
|  | 29.988 | Benzyl Benzoate | 1763 | 1763 | - | - | 1.76 | 1.37 | - | - | 2.7 | - | 5.59 | - | - | 3.04 | 0.91 | 9.33 |
|  | 31.409 | Neophytadiene | 1827 | 1828 | - | - | 2.96 | - | - | - | 0.17 | - | 0.31 | - | - | 0.2 | - | 0.4 |
|  | 31.422 | Benzoic acid, 2-phenylethyl ester | 1805 | 1809 | - | - | - | 0.55 | - | - | - | - | - | - | - | - | - | - |
|  | 31.496 | all-*trans*-Farnesyl acetate | 1832 | 1836 | 0.35 | - | - | - | 3.16 | 1.18 | - | - | 0.24 | 2.21 | 2.27 | - | - | - |
|  | 31.54 | Hexahydrofarnesyl acetone | 1834 | 1835 | - | - | - | - | - | - | - | - | - | - | - | 0.11 | - | 0.29 |
|  | 31.649 | Nerolidyl acetate | 1816 | 1816 | 0.34 | - | - | - | - | - | - | - | - | - | - | - | - | - |
|  | 31.862 | Nerolidyl propionate | 1851 | 1850 | - | - | - | - | 0.2 | - | - | - | - | - | - | - | - | - |
|  | 33.021 | (*Z*)-Methyl hexadec-11-enoate | 1910 | 1913 | - | - | - | - | - | 0.43 | - | - | - | - | 0.8 | - | - | - |
|  | 33.242 | Methyl palmitate | 1921 | 1921 | 0.05 | 15.76 | 1.36 | 3.56 | 0.29 | 3.53 | 1.61 | 0.71 | 1 | - | 5.47 | 2.44 | 1.92 | 2.14 |
|  | 33.661 | Isophytol | 1942 | 1943 | - | - | 0.33 | - | - | - | 5.94 | - | 5.03 | - | - | 10.52 | - | 8.45 |
|  | 34.611 | Ethyl palmitate | 1989 | 1990 | - | - | - | - | 0.15 | - | 0.15 | 0.56 | - | 0.76 | - | 0.32 | 0.9 | - |
|  | 35.24 | Methyl margarate | 2021 | 2021 | - | - | - | - | - | - | - | - | - | - | - | - | 0.06 | - |
|  | 35.342 | Geranyllinalool | 2026 | 2034 | 0.38 | - | - | - | 2.28 | - | 3.01 | - | 1.96 | 6.85 | - | 5.61 | 0.08 | 3 |
|  | 36.47 | (*E*)-Cinnamyl benzoate | 2084 | 2099 | - | - | - | - | 0.22 | - | - | - | - | - | 0.14 | - | - | - |
|  | 36.569 | 9,12-Octadecadienoic acid (*Z*,*Z*)-, methyl ester | 2090 | 2091 | 0.04 | 2.62 | 0.51 | 1.62 | - | 10.36 | - | 1.39 | - | 0.49 | 14.99 | 0.86 | 3.51 | 0.45 |
|  | 36.6 | Heneicosane | 2091 | 2100 | - | - | - | - | 0.49 | - | 0.6 | - | 0.44 | - | - | - | - | - |
|  | 36.71 | 9,12,15-Octadecatrienoic acid, methyl ester, (*Z*,*Z*,*Z*)- | 2097 | 2096 | - | - | - | - | 0.95 | 4.07 | 4.3 | 2.47 | 2.25 | 0.96 | 5.86 | 7.23 | 7.41 | 4.44 |
|  | 36.773 | 9,12-Octadecadien-1-ol, (*Z*,*Z*)- | 2071 | 2069 | - | 11.79 | - | 1.59 | - | - | - | - | - | - | - | - | - | - |
|  | 36.789 | 9-Octadecenoic acid (*Z*)-, methyl ester | 2101 | 2103 | - | - | - | - | - | 1.93 | - | - | - | - | 2.56 | - | - | - |
|  | 36.915 | Phytol | 2108 | 2107 | - | - | - | - | - | - | 5.14 | - | 7.22 | - | - | 9.69 | - | 8.01 |
|  | 36.992 | 11-Octadecenoic acid, methyl ester | 2112 | 2110 | - | - | - | - | - | - | - | - | - | - | 0.45 | - | - | - |
|  | 37.004 | 10-Octadecenoic acid, methyl ester | 2113 | 2110 | - | - | - | - | - | 0.31 | - | - | - | - | - | - | - | - |
|  | 37.172 | Methyl stearate | 2122 | 2122 | - | - | - | 1.08 | 0.34 | 0.78 | - | 0.6 | 0.14 | - | 1.06 | 0.48 | 2.56 | 0.24 |
|  | 37.724 | Linoleic acid | 2152 | 2152 | - | - | - | - | 0.1 | 0.41 | 0.21 | 0.52 | 0.08 | 0.79 | 0.73 | 0.11 | 0.48 | - |
|  | 37.839 | Ethyl linoleate | 2158 | 2159 | - | - | - | - | 0.19 | - | 0.15 | 0.69 | - | 0.6 | 0.19 | 0.3 | 1.09 | 0.06 |
|  | 37.963 | (*Z,Z,Z*)- 9,12,15-Octadecatrienoic acid | 2165 | 2162 | - | - | - | - | 0.76 | - | 0.92 | 1.83 | - | 2.52 | - | 2.02 | 3.1 | - |
|  | 38.097 | Methyl phytenate | 2172 | 2185 | - | - | - | - | - | - | - | - | 0.18 | - | - | 0.3 | - | 0.27 |
|  | 38.352 | (*E*)-Geranylgeraniol | 2189 | 2192 | - | - | - | - | 0.18 | 12.28 | - | - | - | 1.91 | 18.5 | - | - | - |
|  | 38.393 | Ethyl stearate | 2188 | 2188 | - | - | - | - | - | - | - | - | - | - | - | - | 0.08 | - |
|  | 38.854 | Phytol, acetate | 2213 | 2218 | - | - | - | - | - | - | 5.41 | - | 3.81 | - | - | 8.76 | - | 6.63 |
|  | 39.834 | (*Z*)-9-Tricosene | 2268 | 2271 | - | - | - | - | 6.11 | 0.12 | - | - | - | 13.77 | - | - | - | - |
|  | 40.225 | Tricosane | 2290 | 2300 | - | - | - | - | 2.16 | 4.62 | 0.82 | 0.53 | 0.7 | 3.91 | 4.48 | 0.73 | 0.7 | - |
|  | 40.327 | (*E,E,E*)-2,6,10,14-Hexadecatetraen-1-ol, 3,7,11,15-tetramethyl-, acetate | 2296 | 2301 | - | - | - | - | - | 1.77 | - | - | - | - | 2.57 | - | - | - |
|  | 40.393 | 11,14,17-Eicosatrienoic acid, methyl ester | 2300 | 2291 | - | - | - | - | - | - | - | - | - | - | - | 0.05 | - | 0.08 |
|  | 40.477 | (2*E*,6*E*,10*E*)-3,7,11,15-Tetramethylhexadeca-2,6,10,14-tetraen-1-yl formate | 2305 | - | - | - | - | - | - | - | - | - | - | - | - | - | 0.19 | 0.05 |
|  | 40.769 | Methyl eicosanoate | 2322 | 2329 | - | - | - | - | 0.08 | 0.2 | - | 0.06 | - | - | 0.27 | - | 0.32 | - |
|  | 40.813 | *cis*-5,8,11,14,17-Eicosapentaenoic acid | 2325 | 2334 | - | - | - | - | - | - | - | - | - | 1.15 | - | - | - | - |
|  | 41.265 | 4,8,12,16-Tetramethylheptadecan-4-olide | 2351 | 2355 | - | - | - | - | - | - | - | - | 0.08 | - | - | 0.22 | - | 0.18 |
|  | 41.419 | Oleamide | 2360 | 2375 | - | - | - | - | - | - | - | - | - | 1.13 | - | - | - | - |
|  | 41.561 | 1-Tetracosene | 2368 | 2378 | - | - | - | - | 0.35 | - | - | - | - | 1.14 | - | - | - | - |
|  | 41.928 | Tetracosane | 2390 | 2400 | - | - | - | - | 1.08 | 0.21 | - | - | 0.04 | 2.05 | 0.2 | - | - | - |
|  | 41.972 | Hexanedioic acid, bis(2-ethylhexyl) ester | 2392 | 2398 | - | - | - | - | - | - | - | - | - | - | - | - | 0.29 | - |
|  | 42.716 | (2*E*, 6*E*)-Farnesyl benzoate | 2438 | - | - | - | - | - | 0.4 | - | - | - | - | 1.22 | - | - | - | - |
|  | 43.225 | Pentacos-1-ene | 2469 | 2483 | - | - | - | - | 0.07 | - | - | - | - | - | - | - | - | - |
|  | 43.564 | Pentacosane | 2490 | 2500 | - | - | - | - | 3.92 | 2.72 | 0.72 | 0.48 | 0.65 | 4.01 | 1.71 | 0.25 | 0.38 | - |
|  | 44.086 | Methyl docosanoate | 2522 | 2524 | - | - | - | - | 0.06 | 1.26 | - | - | - | - | 1.45 | - | 0.1 | - |
|  | 44.46 | Bis(2-ethylhexyl) phthalate | 2546 | 2546 | - | - | - | - | - | - | - | - | - | - | - | - | - | 0.34 |
|  | 44.714 | 3-Methylpentacosane | 2562 | 2572 | - | - | - | - | - | - | - | - | 0.08 | - | - | - | - | - |
|  | 44.91 | Hexadecanoic acid, phenylmethyl ester | 2575 | 2587 | - | - | - | - | 0.14 | - | - | - | - | - | 0.19 | 0.22 | - | 0.11 |
|  | 45.136 | Hexacosane | 2589 | 2600 | - | - | - | - | 0.19 | 0.12 | 0.12 | 0.08 | 0.12 | - | - | - | - | - |
|  | 46.091 | 2-Methylhexacosane | 2651 | 2656 | - | - | - | - | 0.07 | - | - | 0.27 | 0.05 | - | - | - | 0.24 | - |
|  | 46.653 | Heptacosane | 2689 | 2700 | - | - | - | - | 3.64 | 5.16 | 4.18 | 3.59 | 3.89 | - | 1.23 | 0.47 | 0.54 | - |
|  | 47.165 | Methyl tetracosanoate | 2723 | 2725 | - | - | - | - | 0.25 | 1.08 | - | 0.08 | - | 0.55 | 1.02 | - | 0.11 | - |
|  | 47.461 | 1,3-Benzenedicarboxylic acid, bis(2-ethylhexyl) ester | 2743 | 2730 | - | - | - | - | 0.37 | - | 0.37 | 0.17 | - | 3.07 | 0.49 | 0.54 | 0.42 | - |
|  | 47.57 | 2-Methylheptacosane | 2751 | 2761 | - | - | - | - | - | - | - | 0.13 | - | - | - | - | - | - |
|  | 47.63 | Benzyl linoleate | 2755 | 2764 | - | - | - | - | - | 0.09 | - | - | - | - | 0.32 | - | - | - |
|  | 47.737 | 3-Methylheptacosane | 2762 | 2771 | - | - | - | - | 0.47 | 0.09 | 1.07 | 0.88 | 0.93 | 0.9 | 0.31 | 1.03 | 0.84 | 0.2 |
|  | 48.114 | Octacosane | 2788 | 2800 | - | - | - | - | 0.73 | 0.36 | 0.65 | 0.95 | 0.66 | - | - | - | - | - |
|  | 48.622 | Squalene | 2823 | 2819 | - | - | - | - | 1.41 | 4.16 | 5.62 | 0.82 | 3.86 | 4.44 | 5.66 | 8.13 | 1.18 | 6.52 |
|  | 48.72 | Hexacosanal | 2831 | 2833 | - | - | - | - | - | 0.38 | - | - | - | - | - | - | - | - |
|  | 49.005 | 2-Methyloctacosane | 2851 | 2858 | - | - | - | - | 0.5 | 0.15 | 0.12 | 1.78 | 0.32 | - | - | - | 0.4 | - |
|  | 49.538 | Nonacosane | 2889 | 2900 | - | - | - | - | 11.88 | 8.48 | 14.52 | 19 | 12.55 | - | 0.39 | 0.06 | 0.33 | - |
|  | 50.03 | Hexacosanoic acid, methyl ester | 2924 | 2929 | - | - | - | - | 0.16 | 0.37 | 0.14 | - | 0.07 | - | 0.2 | - | - | - |
|  | 50.296 | 2,3-epoxysqualene | 2944 | 2963 | - | - | - | - | 1.12 | 3.27 | 12.03 | 33.17 | 8.55 | 1.32 | 4.96 | 20.98 | 49.4 | 13.25 |
|  | 50.546 | 3-Methylnonacosane | 2962 | 2958 | - | - | - | - | 1.42 | 0.54 | 2.91 | 2.6 | 2.8 | - | 0.22 | 0.44 | 1 | - |
|  | 50.893 | Triacontane | 2987 | 3000 | - | - | - | - | 1.52 | 0.47 | 1.23 | 0.9 | 1.09 | - | - | - | - | - |
|  | 50.931 | Benzyl icosanoate | 2990 | 3003 | - | - | - | - | - | - | - | - | - | - | - | 0.35 | - | 0.19 |
|  | 51.106 | Hexacosyl acetate | 3003 | 3010 | - | - | - | - | - | 0.6 | - | - | - | - | 0.45 | - | - | - |
|  | 51.286 | 1,6,10,14,18,22-Tetracosahexaen-3-ol, 2,6,10,15,19,23-hexamethyl-, (all-*E*)-(+/-)- | 3017 | 3030 | - | - | - | - | - | - | - | - | - | - | - | - | 0.15 | 0.1 |
|  | 51.51 | Octacosanal | 3034 | 3032 | - | - | - | - | - | 0.29 | - | - | - | - | - | - | - | - |
|  | 51.726 | 2-Methyltriacontane | 3050 | 3059 | - | - | - | - | 1.46 | 0.79 | 0.22 | 1.72 | 0.34 | - | - | - | - | - |
|  | 52.238 | Hentriacontane | 3089 | 3100 | - | - | - | - | 22.67 | 8.66 | 11.75 | 12.32 | 9.98 | - | - | - | - | - |
|  | 52.429 | Heptacosyl acetate | 3103 | 3112 | - | - | - | - | - | 0.26 | - | - | - | - | - | - | - | - |
|  | 52.709 | Octacosanoic acid, methyl ester | 3125 | 3125 | - | - | - | - | 0.14 | 0.25 | 0.36 | 0.08 | 0.2 | - | - | - | - | - |
|  | 52.919 | Vitamin E | 3141 | 3142 | - | - | - | - | - | 0.17 | - | 0.14 | - | - | 0.16 | - | - | - |
|  | 53.177 | Hentriacontane, 3-methyl- | 3160 | 3169 | - | - | - | - | 1.23 | 0.65 | 1.63 | 1.03 | 1.8 | - | - | - | - | - |
|  | 53.51 | Dotriacontane | 3186 | 3200 | - | - | - | - | 1.14 | 0.27 | 0.36 | 0.36 | 0.35 | - | - | - | - | - |
|  | 53.67 | Farnesyl palmitate | 3198 | 3204.1 | - | - | - | - | 0.37 | 0.41 | 0.27 | 0 | 0.22 | - | - | - | - | - |
|  | 53.737 | Octacosyl acetate | 3203 | 3215 | - | - | - | - | - | 1.23 | - | - | - | - | 0.33 | - | - | - |
|  | 54.376 | Dotriacontane, 2-methyl- | 3248 | 3254 | - | - | - | - | 0.56 | 0.24 | - | 0.63 | 0.09 | - | - | - | - | - |
|  | 54.549 | 3-Methyldotriacontane | 3260 | 3273 | - | - | - | - | - | - | - | - | 0.05 | - | - | - | - | - |
|  | 54.931 | Tritriacontane | 3286 | 3276 | - | - | - | - | 3.96 | 1.45 | 1.59 | 1.42 | 1.53 | - | - | - | - | - |
|  | 55.785 | gamma-Sitosterol | 3340 | 3351 | - | - | - | - | 0.4 | 0.24 | - | 0.24 | - | 1.51 | 0.56 | 0.29 | 0.53 | - |
|  | 56.129 | Tritriacontane, 3-methyl- | 3360 | 3376 | - | - | - | - | 0.53 | 0.24 | 1.42 | 0.39 | 1.19 | - | - | - | - | - |
|  | 56.505 | *α*-Amyrin | 3383 | 3376 | - | - | - | - | - | - | - | - | - | - | 0.49 | - | 0.69 | - |
|  | 56.881 | Triacontyl acetate | 3418 | 3412 | - | - | - | - | - | 0.59 | - | - | 0.6 | - | - | - | - | - |
|  | 58.445 | Hexatriacontane | 3580 | 3600 | - | - | - | - | 0.15 | - | - | - | - | - | - | - | - | - |
|  | 59.791 | Phytyl heptadecanoate | 3673 | 3646 | - | - | - | - | - | - | - | - | - | - | - | 1.15 | - | 1.36 |
|  |  | **Monoterpene hydrocarbons** |  |  | **1.57** | **1.44** | **0.26** | **1.76** | **0** | **0** | **0** | **0** | **0** | **0** | **0** | **0** | **0** | **0** |
|  |  | **Oxygenated monoterpenes** |  |  | **25.52** | **3.37** | **38.19** | **0** | **0.73** | **0** | **0** | **0** | **1.76** | **2.09** | **0** | **0** | **0** | **4.35** |
|  |  | **Sesquiterpene Hydrocarbons** |  |  | **12.29** | **12.82** | **2.73** | **13.54** | **1.24** | **0** | **0.72** | **1.16** | **1.13** | **0** | **0** | **0.8** | **1.8** | **2.19** |
|  |  | **Oxygenated sesquiterpenes** |  |  | **1.69** | **0** | **0** | **37.81** | **16.11** | **11.09** | **0.27** | **3.99** | **0.46** | **25.69** | **19.31** | **0.11** | **12.13** | **0.4** |
|  |  | **Diterpenes** |  |  | **0.38** | **0** | **3.29** | **0** | **2.86** | **14.05** | **19.67** | **0** | **18.33** | **9.98** | **21.07** | **34.78** | **0.27** | **26.54** |
|  |  | **Triterpene** |  |  | **0** | **0** | **0** | **0** | **2.53** | **7.43** | **17.65** | **33.99** | **12.41** | **5.76** | **11.11** | **29.11** | **51.42** | **19.87** |
|  |  | **Phenylpropanoids/Benzenoids** |  |  | **16.73** | **36.51** | **30.79** | **8.66** | **1.02** | **0.09** | **2.7** | **0** | **15.76** | **0** | **0.46** | **4.44** | **1.27** | **29.79** |
|  |  | **Fatty-acid derivatives** |  |  | **40** | **30.17** | **19.35** | **29.66** | **3.61** | **27.66** | **8.69** | **8.99** | **6.06** | **8.95** | **36.02** | **17.46** | **22.03** | **13.13** |
|  |  | **Aliphatic hydrocarbons** |  |  | **0** | **0** | **0** | **0** | **66.3** | **35.34** | **43.91** | **48.81** | **39.65** | **25.78** | **8.54** | **2.98** | **4.43** | **0.2** |
|  |  | **Nitrogen-Containing Compounds** |  |  | **0.03** | **3.55** | **0.98** | **0** | **0** | **0** | **0** | **0** | **0.47** | **0** | **0** | **0** | **0** | **0.71** |
|  |  | **Others** |  |  | **0** | **4.35** | **0** | **4.39** | **0.77** | **1.08** | **0.37** | **0.55** | **0** | **4.58** | **1.21** | **1.17** | **1.24** | **0.43** |
|  |  | **Total identified compounds (%)** |  |  | **98.21** | **92.21** | **95.59** | **95.82** | **95.17** | **96.74** | **93.98** | **97.74** | **96.03** | **82.83** | **97.72** | **90.85** | **94.59** | **97.61** |

**A**

**B**

**
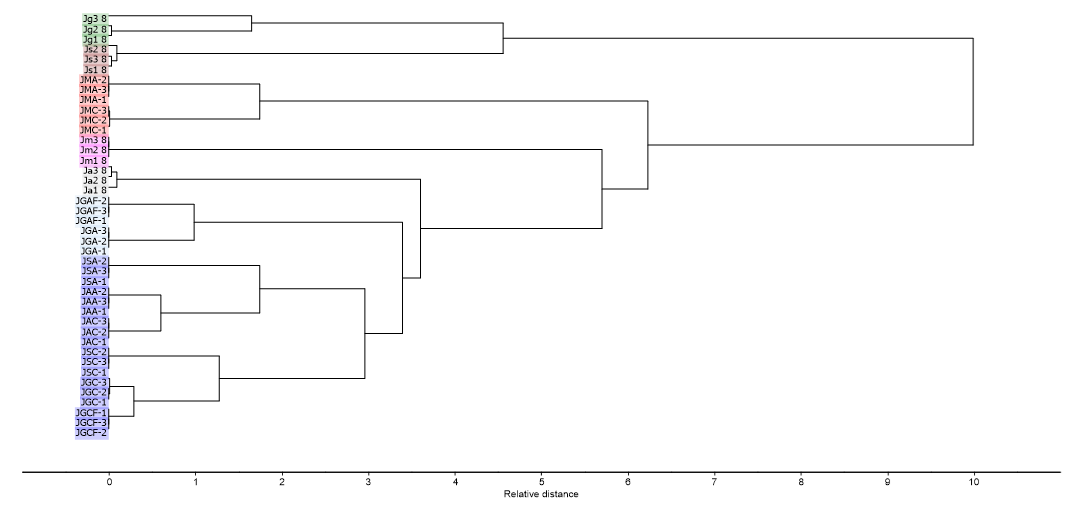
**

**C**

**1a**

**1b**

**Fig.** **S1.** Principal component analysis of DHS of *Jasminum* species collected in August combined with jasmine concrete and absolute composition as analyzed by GC-MS: (A) A score plot and (B) Loading plot for PC1 and PC2 with associated contributing metabolites. (C) Dendrogram obtained from Hierarchical Cluster Analysis (HCA).

**A**

**B**

**Fig. S2.** Principal component analysis of seasonal fluctuation in *Jasminum sambac* DHS using GC-MS: (A) Score plot and (B) Loading plot for PC1 and PC2 with contributing metabolites.

**A**

**B**

**Fig. S3.** Principal component analysis of seasonal fluctuation in *Jasminum azoricum* HS using GC-MS: (A) Score plot and (B) Loading plot for PC1 and PC2 with contributing metabolites.

**A**

**B**

**Fig. S4.** Principal component analysis of seasonal fluctuation in *Jasminum grandiflorum* HS using GC-MS: (A) Score plot and (B) Loading plot for PC1 and PC2 with contributing metabolites.

**A**

**B**

**Fig. S5.** Principal component analysis of seasonal fluctuation in *Jasminum multiflorum* HS using GC-MS: (A) Score plot and (B) Loading plot for PC1 and PC2 with contributing metabolites.

**Fig. S6.** Dose response curves of four jasmine species (concrete) (A) *J. grandiflorum* concrete, (B*) J. grandiflorum* Factory concrete, (C) *J. sambac* concrete, (D) *J. azoricum* concrete and (E) *J. multiflorum* concrete. All determinations were performed in triplicate. The mean and standard deviation are used to express the values.

**Fig. S7.** Dose response curves of four jasmine species (absolute) (A) *J. grandiflorum* absolute, (B*)* purchased *J. grandiflorum* absolute, (C) *J. sambac* absolute, (D) *J. azoricum* absolute and (E) *J. multiflorum* absolute. All determinations were performed in triplicate. The mean and standard deviation were used to express the values.

**Fig. S8.** Variable Importance in the Projection (VIP) plot demonstrating those components that contributed the most to the biological activity
